# Mechanical upside of PAO mainstream fixations: co-simulation based on early postoperative gait characteristics of DDH patients

**DOI:** 10.3389/fbioe.2023.1171040

**Published:** 2023-07-19

**Authors:** Peng Yang, Qi Liu, Tianye Lin, Aobulikasimu Aikebaier, Luoyong Jiang, Weichao Sun, Qingwen Zhang, Xueling Bai, Wei Sun

**Affiliations:** ^1^ Department of Orthopedics, Shenzhen Second People’s Hospital, Shenzhen, Guangdong, China; ^2^ Shenzhen Institute of Advanced Technology, Chinese Academy of Sciences (CAS), Shenzhen, Guangdong, China; ^3^ Traumatology and Orthopedics Institute of Chinese Medicine of Guangdong, Guangzhou, Guangdong, China; ^4^ Guangdong Provincial Hospital of Chinese Medicine, Guangzhou, Guangdong, China

**Keywords:** musculoskeletal simulation, finite element, periacetabular osteotomy, developmental dysplasia of the hip, gait analysis

## Abstract

**Purpose:** To investigate the early postoperative gait characteristics of patients who underwent periacetabular osteotomy (PAO) and predict the biomechanical performance of two commonly used PAO fixation methods: iliac screw (IS) and transverse screw (TS).

**Methods:** A total of 12 patients with unilateral developmental dysplasia of the hip (DDH) (mean age 27.81 ± 4.64 years, 42% male) that were scheduled to undergo PAO surgery were included in this study. Their preoperative CT images and pre- and postoperative gait data were used to create subject-specific musculoskeletal models and complete the inverse dynamics analysis (IDA). Two patients with typical gait characteristics were selected using clustering analysis, and their IDA data were incorporated into finite element (FE) models of IS and TS fixations. Failure simulation was performed by applying iterative steps with increasing gait load to predict yield load. Stress results and yield loads were calculated for each FE model at different phases of the gait cycle.

**Results:** Postoperative gait showed improvement compared to preoperative gait but remained inferior to that of healthy individuals. Postoperative gait was characterized by a lower hip range of motion, lower peri-ilium muscle forces, particularly in the abductors, and a sharper initial peak and flatter second peak of hip joint reaction force (HRF). Finite element analysis (FEA) showed a trend of increasing stress during the second–fourth phases of the gait cycle, with lower stress levels in other phases. At high-stress gait phases, the mean stress of maximum ${\bar{p}}_{100}$ differed significantly between IS and TS (*p* < 0.05) and between coupled and uncoupled muscle forces (*p* < 0.05). Failure analysis predicted a slightly larger yield load for TS configurations (6.21*BW) than that for IS (6.16*BW), but both were well above the gait load. Coupled and uncoupled groups showed similar results, but uncoupled groups had lower yield loads (5.9*BW).

**Conclusion:** PAO early postoperative gait shows a normalized trend, but abnormalities persist. IS and TS are both capable of resisting mechanical strain failure, with no significant mechanical advantage found for transverse screw fixation during PAO early postoperative gait. Additionally, it is important to note that the TS may have a higher risk of cyclic fatigue failure due to the localized greater stress concentration. Furthermore, the most medial screw is crucial for pelvic stability.

## Introduction

Developmental dysplasia of the hip (DDH) is a complex pathology affecting the acetabulum or femoral head and a leading cause of secondary hip osteoarthritis. Various salvage methods, such as Chiari pelvic osteotomy, acetabular rotation osteotomy, and periacetabular osteotomy (PAO), are commonly used in the therapy of DDH to prevent or postpone secondary osteoarthritis (Ito et al., 2011a; Yasunaga et al., 2017). Among them, PAO, which can preserve the patient’s own hip, improve the consistency, and mediate the rotation center of the hip joint by rotating and moving the acetabular fragment during operation, has been shown to be a required surgical method for treating DDH in adolescents and young adults up to 40 years old. Numerous studies have reported favorable clinical, functional, and radiological outcomes, as well as medium- to long-term survival rates for preserved hip joints (Ito et al., 2011b). The polygonal shape of the acetabular fragment and the integrity of the acetabular posterior column part with the surrounding muscles and other soft tissues can provide initial stability to the ilium, so only minimal screws are needed for fixation in PAO (Mechlenburg et al., 2007). However, the fixation of the reorientation acetabular fragment remains a challenge. It has also been shown to have some postoperative complications such as the non-union of one of the osteotomies and stress fracture, which may be related to the internal fixation (Malviya et al., 2015; Leopold et al., 2021; Morris et al., 2022). However, two common screw internal fixation techniques have been reported: 1) iliac screw (IS) fixation, in which three cortical screws of different lengths are inserted from the iliac crest into the acetabular fragment, and 2) transverse screw (TS) fixation, in which two cortical screws are inserted from the iliac crest into the acetabular fragment, and then, an additional cortical screw is inserted transversely from the acetabular fragment into the ilium.

Both IS and TS fixation techniques are widely utilized in clinical practice. The IS technique involves implanting screws in a direction from above the iliac crest downward, allowing for rapid fixation of the acetabular fragment. This facilitates intraoperative fluoroscopic assessment of screw placement and reduces surgical time. On the other hand, the TS technique requires additional transverse screw insertion, which may increase the difficulty of screw implantation due to potential interference between screws, thereby prolonging the surgical duration. Additionally, the inconsistent screw entry points in the TS technique necessitate larger skin incisions during subsequent screw removal procedures, leading to greater surgical trauma. However, some researchers argue that the TS technique, employing dual-plane fixation and multipoint support, provides better resistance against shear forces and enhances the overall pelvic stability.

PAO patients are suggested to benefit from rehabilitation training starting on the first postoperative day and make a positive transition from aided walking to unaided walking with the affected side bearing full weight postoperatively after 3–6 months (Disantis et al., 2022). The defining features of early postoperative gait of patients undergoing PAO remain unknown, and the difference between the biomechanical performance of IS and TS fixations under these circumstances needs to be studied yet. Simulation-based techniques, such as finite element analysis (FEA), offer valuable insights into the field of biomechanics. These methods have been utilized to investigate the force transmission mechanism and mechanical behavior of various screw fixation techniques in PAO^9^. Although these studies have been informative, there are limitations to them. Individual muscles that should contribute to the deformation and stress of the skeleton were not represented in these models, and static forces were usually used as inputs to the simulation, which may not be able to reflect the natural loading conditions of human physical activities. In a previous study, we constructed and validated a co-simulative framework based on the subject-specific musculoskeletal-multibody dynamic (MSK-MBD) model and deformable finite element (FE) model, which have also been used to analyze the mechanical behavior of the hip joint during the normal gait (Xiong et al., 2022). This study elucidates the characteristics of muscular force responses during gait and their impact on joint reaction force, highlighting the significance of high-stress gait phases in hip biomechanical analysis. It provides possibilities for investigating the distinctive features of various gait patterns and conducting individualized mechanical simulations from a biomechanical perspective.

Based on the aforementioned ideas, the objective of this study was to construct a specialized co-simulation framework that integrates early postoperative gait characteristics and skeletal geometrical morphology parameters of patients, specific to periacetabular osteotomy. The purpose of this framework is to forecast the biomechanical efficacy of various internal fixation methods during the early postoperative gait phase.

## Methods

### Data collection

This study was carried out in conformity with the Declaration of Helsinki and prevailing ethical principles. Our study was approved by the Ethics Committee of Shenzhen Second People’s Hospital (No. 20220802012). The selection of cases was based on the following criteria:

Inclusion criteria:

① Unilateral hip dysplasia requiring PAO (with Crowe’s classification of grade I or II, Hartofilakidis classification of grade I, and Tönnis stage I or II);
② Age between 18 and 45 years.

Exclusion criteria:

① History of previous lower limb surgery;
② Neurological disorders;
③ BMI >30 kg/m^2^;
④ Development of severe postoperative complications that impede the completion of rehabilitation training;
⑤ Coexistence of other conditions affecting the gait;
⑥ Presence of other contraindications for surgery.

A total of 12 patients were included in this study (Table 1). Informed consent was obtained from all subjects. All PAO operations were performed by the same experienced surgeon who successfully passed through his learning curve (performing more than 20 PAO operations).

**TABLE 1:** Clinical characteristics of patients.

Characteristic	Value
Age	27.81 ± 4.64 years
Sex	
Female	7
Male	5
BMI	21.52 ± 2.7 kg/m^2^
Surgical side	
Right	6
Left	6
CE degree	28.22 ± 2.51°
Crowe’s classification	I–II
Hartofilakidis classification	I
Tönnis stage	I–II

Data are presented as the mean ± SD or numbers. BMI, body mass index; CE, central-edge angle.

#### Imaging data

**FIGURE 1:**
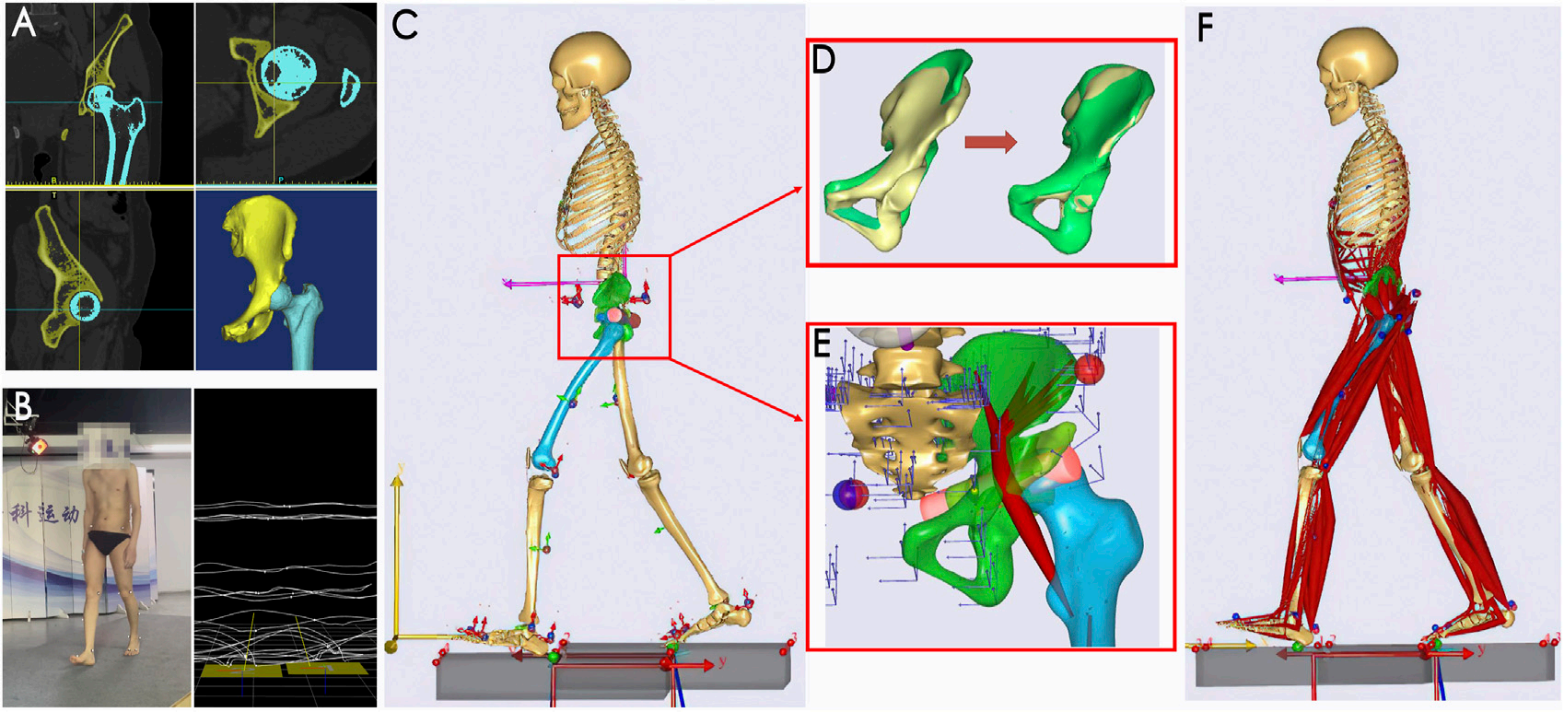
Subject-specific MSK-MBD modeling. **(A)** 3D reconstruction; **(B)** gait data acquisition; **(C)** MBD model with individualized segments; **(D)** morphological scaling of the ilium segment; green represents the general ilium, and yellow represents the subject’s ilium; **(E)** optimization of the muscle cylindrical wrapping surface to fit the reorientated acetabular fragment; **(F)** MSK model with individualized settings. MSK-MBD, musculoskeletal-multibody dynamic.

Preoperative anteroposterior X-ray radiographs of the pelvis were obtained to assess the bony structure. The volume CT images of the ilium–femur were obtained using a CT scanner (slice thickness 2 mm and retrospectively reconstructed to 5 mm; slice spacing 2 mm; resolution 1,024 × 1,024). The pelvic bony region was identified by applying threshold-based segmentation and manual refinement, and then, the 3D pelvic model was reconstructed using Mimics software (version 16.0, Materialise, Belgium), as shown in Figure 1A.

#### Gait data

Kinematic and kinetic data were obtained during walking using the optical motion capture system (BTS Bioengineering, Italy), both preoperatively and 6 months postoperatively. Prior to gait data collection, all patients underwent rehabilitation training and resumed their daily activities. During the tests, the patients were instructed to perform adaptive gait training prior to attaching 22 markers to their lower extremities based on bony landmarks. They were then asked to perform the formal walk tests (Figure 1B). Five rounds of walking data were collected for each patient, including ground reaction moments and forces, using two ground reaction force plates at 1,000 Hz (BTS P-6000, Italy). Three typical gait data were identified based on clustering analysis in MATLAB software (R2017a, MathWorks, United States); then, these data were outputted in the C3D file format for subsequent procedures.

### Subject-specific MSK-MBD simulation

**FIGURE 2:**
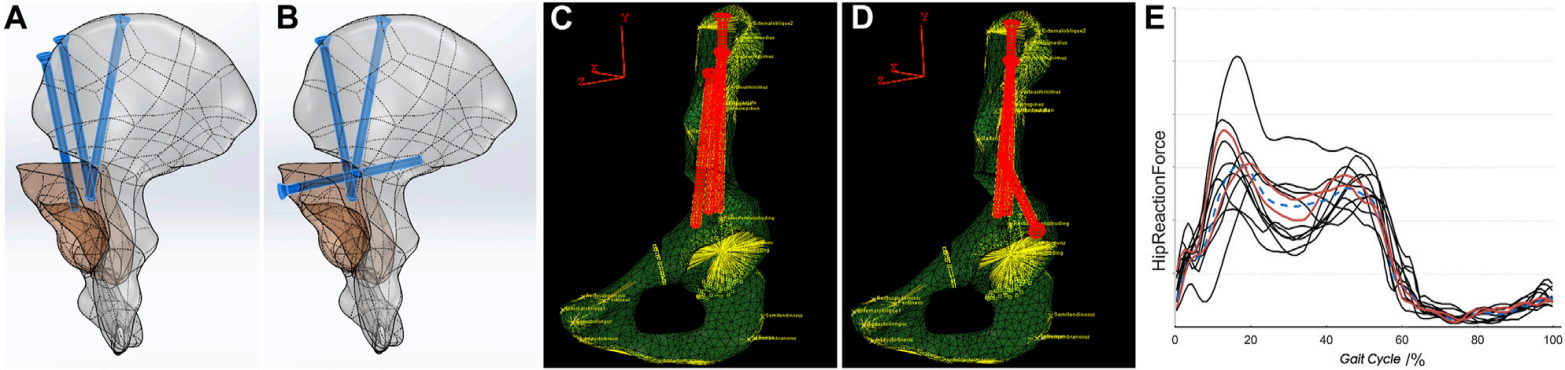
PAO simulation and FE modeling. **(A,B)** PAO simulation using different fixation techniques; **(C,D)** FE modeling with the coupling peri-ilium muscles; **(E)** clustering based on the HRF results; red curves represent the two screened subjects. PAO, periacetabular osteotomy; FE, finite element; HRF, hip joint reaction force.

PAO simulation: PAO surgery simulation was performed based on the preoperative pelvic model in SolidWorks (version 2020, SolidWorks Corporation, United States). The osteotomy procedures including acetabulum resecting and screw implanting were simulated as described in Ganz et al. (1988); then, a dissociated acetabular fragment was created, rotated, and moved inwardly to make the fragment fit into a satisfactory position (Figures 2A, B).

MBD simulation: Subject-specific MSK-MBD simulation was performed in the AnyBody modeling system (version 7.1, AnyBody Technology, Denmark) using the general lower extremity body model (Figure 1C). While the basic function and application of AnyBody technology have been well introduced previously (Damsgaard et al., 2006), we focused on the customization process. First, a general lower extremity model was scaled with individual body mass and body fat; the scaling formula was given as follows:

$$F=F_{0}\frac{k_{m}}{k_{l}}\frac{R_{muscle,1}}{R_{muscle,1}},R_{muscle}=0.5-R_{fat},$$ (1)

where F and F_0_ are the subject’s MBD model maximum muscle strength; *k*_*m*_ and *k*_*L*_ are the ratio of body mass and height of the MBD model, respectively; and *R*_*muscle*_ and *R*_*fat*_ are the ratio of muscle and fat, respectively. A secondary morphological scaling of the ilium of the MBD model was performed by referring to the individual ilium CT model. During the scaling, the insertion points of the peri-ilium muscles in the MBD model were modified automatically to match the geometric shape of the patient’s ilium (Figure 1D). Then, the reorientated acetabular fragment simulated in SolidWorks was introduced into the MBD model separately, and a new rotation center of the hip in the MBD model was redefined to coincide with the fitting sphere center of the acetabular fragment. The iliopsoas muscle was refined as the wrapping muscle with a wrapping surface at the superior of the pubis–acetabulum. By defining the wrapping surface as an independent cylinder, it can be adjusted to be better fit into the reorientated acetabular fragment (Figure 1E). The individual gait data were imported into the subject-specific MBD model to perform kinematic optimization by using the built-in algorithm. After the optimization, the MBD model was driven to perform the corresponding walking actions, and the kinematic data were collected.

MSK simulation and IDA: Inverse dynamic analysis (IDA) was performed based on the dynamic data, including ground reaction moments and forces, as well as the kinematic output data, to drive the MSK model, which contained the Hill muscle simulation model with the following assigned muscle recruitment criterion (Figure 1F). The muscle recruitment problem was formulated as an optimization problem and solved by minimizing a cubic polynomial cost function (de Zee et al., 2007), which was given by

$$G\left(f_{i}^{\left(M\right)}\right)=\sum_{i}^{n^{\left(M\right)}}{\left(\frac{f_{i}^{\left(M\right)}}{N_{i}}\right)}^{p},p\geq 1,$$ (2)

$$Cf=r,f_{i}^{\left(M\right)}\geq 0,$$ (3)

where *G* is the hypothetical muscle force distribution strategy of the central nervous system; *Ni* is the tensile strength of each muscle under the current working condition; *f(M)* is the muscle force that balances the external loads; $f_{i}^{\left(M\right)}$ is the *i*th muscle force; the value of *p* adjusts the power series of the polynomial; *C* represents the coefficient matrix of the equation; *r* is the vector of the external and inertia force; and *f* is the vector of the muscle and joint force. A specifically designed structure was used in the MSK model by introducing the reorientated acetabular fragment. The new RCH of the MSK model was defined to coincide with the fitting sphere center of the acetabular fragment.

Each patient was guaranteed to have three separate typical gait data to drive the MSK-MBD model to complete the kinematic analysis and IDA. The output data of simulation, including kinematics in the hip joint range of motion (RoM) and inverse dynamics in the hip joint reaction force (HRF) and peri-ilium muscle forces, were normalized to a gait cycle (0%–100%) and then expressed as curves in the form of the mean and SD. Furthermore, the gait cycle was divided into eight phases based on RLA classification (Gronley and Perry, 1984) to facilitate subsequent FE modeling.

## FE simulation

### Boundary and loading conditions

Two subjects of different genders were screened using the clustering algorithm based on the HRF results (Simonsen, 2014) (Figure 2E), and their PAO simulation models were introduced into Abaqus software (version 6.8, Simulia, United States) for FE modeling. A new Cartesian coordinate system was redefined, referring to the local coordinate system of the pelvis in the MBD model. The origin of the coordinate system was set at the midpoint of the posterior superior iliac spine, with the XY plane passing through the midpoint of the pubic symphysis and perpendicular to the ground and the XZ plane parallel to the ground. Therefore, the output data of the IDA of eight gait phases could be referenced in the coordinate system and coupled to the corresponding area of the ilium surface in the form of three components as the FE boundary condition setting (Figures 2C, D). This process was automated using the AnyBody plugin called the “AnyFE Converter Tool for Abaqus” (detailed information is available at: https://www.anybodytech.com/resources/customer-downloads/). According to the contact method described in the reference, the threaded screw area was used for binding to the bone (He et al., 2022). According to the report of Suzuki et al. (2020), meshes that included a 1-cm margin surrounding the osteotomy surface were degraded with weakening of the mesh material and simulated a callus during the bone healing process*.*

### Material properties

It was defined that the bone and screw were all continuous, homogeneous, and isotropic linearly elastic. The elastic modulus and Poisson’s ratio of the pelvis bone were determined based on CT density values using a standardized equation for determining the bone mineral density (BMD, ρ in g/cm^3^) from the Hounsfield unit (HU) (Wang et al., 2021a). The apparent BMD was computed using a standardized equation as follows:

$$\rho \;\left(g/\;{cm}^{3}\right)=\left[HU\;\pm \;1.4246\right]\times \frac{0.001}{1.058}\;\left[HU\;value\;>-1\right],$$ (4)

$$\rho \;\left(g/\;{cm}^{3}\right)=0\;\left[HU\;value\leq -1\right].$$ (5)

### Convergence analysis

The grid convergence analysis was performed on finer mesh to validate the accuracy and confidence of the simulations (Wang et al., 2021b; Kitamura et al., 2021; Maslov et al., 2021; Hedelin et al., 2022). According to the literature, an initial mesh size of 4 mm was selected and subsequently refined by 0.5 mm. The adequacy of the mesh was evaluated by examining the maximum von Mises stress results for each mesh iteration, with the criteria for mesh selection defined as a maximum von Mises stress difference between meshes to be within 1%. The mesh convergence analysis, specifically for the FE model in the second gait phase under boundary loading conditions for the first subject, is presented in Table 2. Consequently, the mesh from the third refinement step was chosen for each model.

**TABLE 2:** Mesh convergence analysis for the model at gait phase 2

	Initial mesh	One-step refinement	Two-step refinement	Three-step refinement	Four-step refinement
Size	4 mm	3.5 mm	3 mm	2.5 mm	2 mm
Number of meshes					
Maximum stress	213.9 MPa	209.4 MPa	206.1 MPa	205.3 MPa	205.3 MPa
Stress variation rate	—	2.1%	1.6%	<0.1%	<0.1%

### Failure simulation

The literature reveals that the maximum principal strain criterion is appropriate to judge mesh failure and predict yield load (Koivumäki et al., 2012; Albogha et al., 2016). An incremental protocol starting at the maximum load gait phase and increasing at increments of 10 N was implemented to repeat the FE simulation and recompute the number of failed meshes until it reached 1% of the total volume (Pistoia et al., 2002). The load at that point was considered the model’s yield load subject to gait load.

Preoperative and postoperative kinematics and IDA results of the 12 patients were normalized to the gait cycle (0%–100%) and compared to those of healthy volunteers as previously published. The FE models of two selected patients at eight gait phases were assigned to the coupled muscle force group and uncoupled muscle force group, and the biomechanical performance of IS and TS internal fixation techniques was compared. The maximum top 100 von Mises stress at integration points was extracted to calculate the mean ${\bar{p}}_{100}$ , which could be considered the mean peak stress*.* A weighted mean value of stress (WMV_s−100_) was calculated as follows:

$${WMV}_{s-100}=\sum_{i=1}^{8}{\bar{p}}_{100-i}\frac{p_{i}}{S_{p}},$$ (6)

where $p_{i}$ is the peak stress in the *i*th phase and $S_{p}$ is the sum of $p_{i}$ in the eight phases.

Furthermore, to better compare the mechanical effects of the TS and IS techniques, two-screw (2S) fixation models were designed and simulated for failure analysis. The 2S model involved the use of only two screws for fixation, achieved through a combination and permutation method.

## Results

**FIGURE 3:**
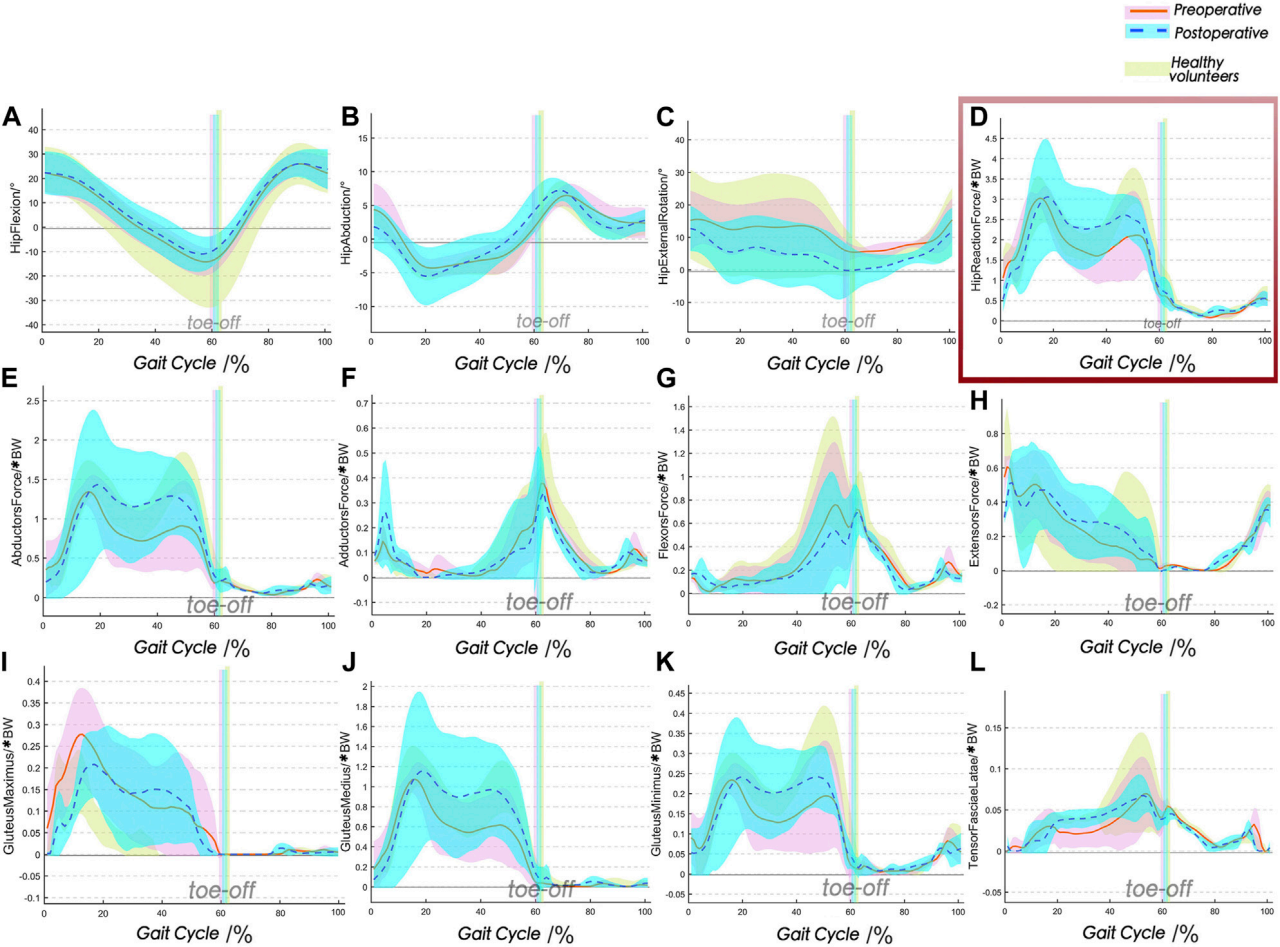
Comparison between preoperative, postoperative, and healthy groups for the kinematics and IDA during gait (mean ± SD). The postoperative data of PAO demonstrate a normalized trend, but abnormalities persist. **(A–C)** Kinematic results; **(D)** HRFs; **(E–H)** peri-ilium muscle forces are grouped as extensors, flexors, abductors, and adductors; **(I–L)** principal components of abductors. HRF, hip joint reaction force.

The subject-specific MSK-MBD models were successfully utilized, and the findings related to kinematics and IDA are presented herein. As shown in Figures 3A–C, the RoM of the hip joint in the three planes showed similar patterns, albeit with variations in amplitude between groups. The patients demonstrated improved RoM of the hip joint from the preoperative stage to 6 months after PAO in terms of external–internal rotation (8.4° ± 3.3°, 13.6° ± 4.7°) and abduction–adduction motion (11.5° ± 1.8°, 14.3° ± 2.1°), whereas flexion–extension motion demonstrated inferior results (37.6° ± 5.4°, 35.5° ± 7.8°). These showed considerably lower RoM of the hip joint than those of the healthy group in preoperative and postoperative groups. The output results of IDA were normalized to subject body weight (BW). As shown in Figure 3D, the curves of HRF in the healthy group were of “M” shape with double peaks, whereas the “M” shape in the PAO group was less pronounced. This suggests that the initial peaks were sharper and steeper with more significant prominence in the postoperative group (3.12*BW ± 1.41*BW), while the second peaks were lower, flatter, and more prominent in the preoperative group (2.11*BW ± 1.03*BW). Additionally, the peri-ilium muscles were classified based on their function and activity, whereby the isogroup muscles were activated during the closing gait phases, and the peak gait phases were consistent. However, there were amplitude variances observed (Figures 3E–H). The PAO groups presented overall lower trends compared to the healthy group, especially among flexor forces and abductor forces. The comparison between preoperative and postoperative groups revealed more noticeable differences in flexors and abductors. The average peak flexor force in the preoperative group was 0.73*BW ± 0.52*BW, compared to 0.54*BW ± 0.47*BW in the postoperative group. Similarly, the average peak abductor force in the preoperative group was 1.32*BW ± 0.38*BW, whereas it was 1.45*BW ± 0.82*BW in the postoperative group. Overall, the abductors had the highest proportion of peri-ilium muscle forces, and more specific assignments for individual muscles are shown in Figures 3I–L.

**FIGURE 4:**
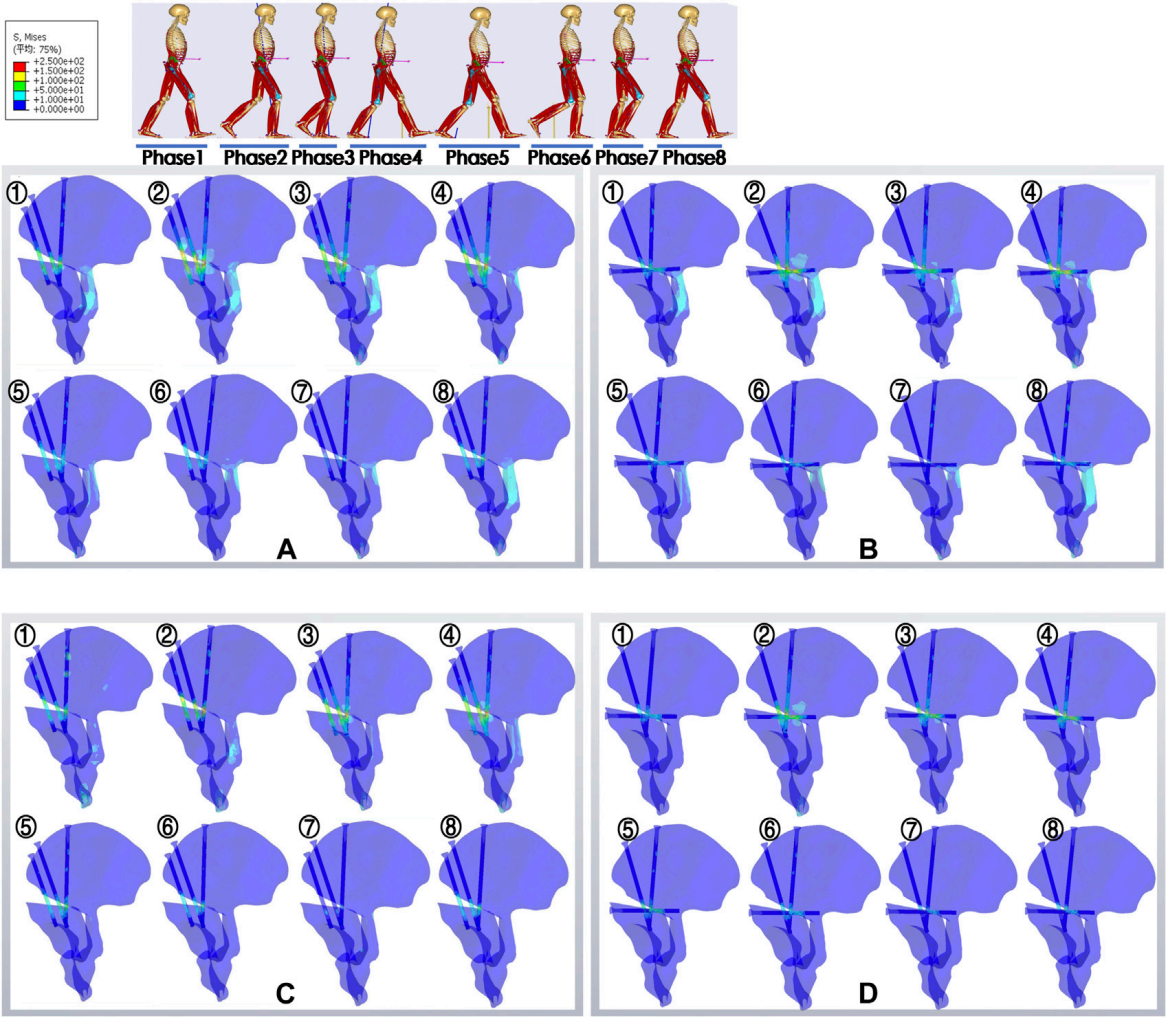
Nephograms of von Mises stress in different groups at eight gait phases. The stress distribution of each model and its variation throughout the gait phases are presented. Highlighted areas indicate stress concentration. ①–⑧ are representative of phases 1–8; **(A,B)** IS and TS groups with the coupling muscle forces; **(C,D)** IS and TS groups without the coupling muscle forces. IS, iliac screw; TS, transverse screw.

The von Mises stress profiles were outputted and are presented in Figure 4; the color changed from red to blue, representing the stress variation. The stress profiles exhibited good agreement between the two subjects. The stress distribution and its variations during the gait cycle were compared between the IS and TS groups. The results indicated that there was little difference between the two groups in terms of stress distribution and changes observed during the gait cycle. Specifically, both groups exhibited higher stress levels during the second–fourth gait phases and lower stress levels during the other phases. These findings were consistent with the variation trends observed in the HRFs of IDA, indicating that the models of both groups responded appropriately to gait load. Moreover, stress concentration areas were predominantly located around the osteotomy site above the acetabular fragment. These areas corresponded to the lower-middle segments of screws and the nail holes of the bone, with a more pronounced effect observed at the most medial screws of the two groups. The coupled muscle force group and uncoupled muscle force group were compared, and the *cloud* diagram showed that the high-stress areas in the uncoupled group were expressed as ribbon-shaped at the middle segments of the screws, which meant more constrictive *concentration* areas and higher peak stress value. Furthermore, the obvious light blue clouds referring to moderate stress were plotted at the acetabular posterior column of the coupled group models in each gait phase, whereas those of uncoupled group models were not plotted.

**FIGURE 5:**
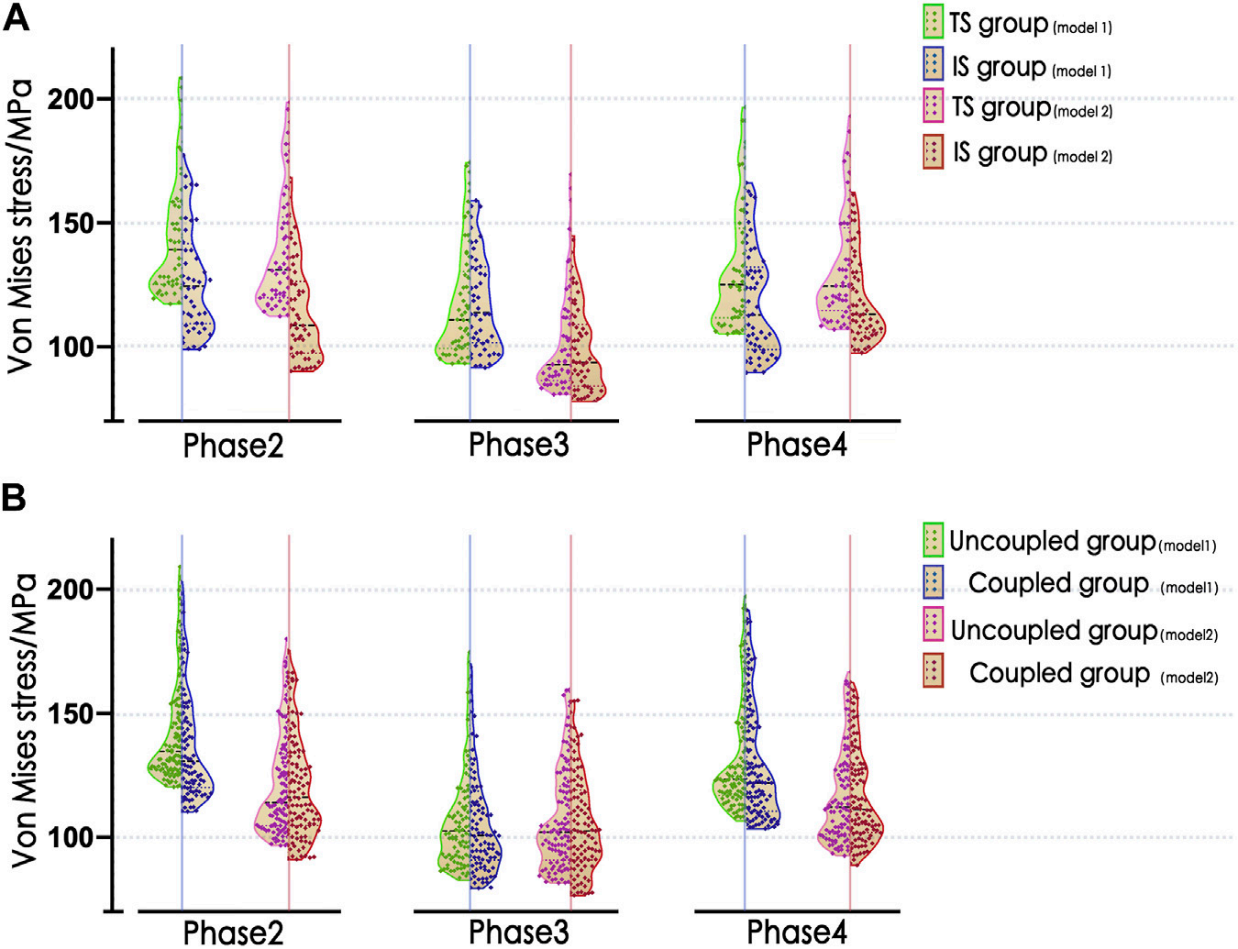
Violin plots of the distribution of ${\bar{p}}_{100}$ values at the high-stress phase. **(A)** TS vs. IS group; **(B)** uncoupled vs. coupled group. IS, iliac screw; TS, transverse screw; uncoupled group, uncoupled muscle force group; coupled group, coupled muscle force group; ${\bar{p}}_{100}$ , mean of the maximum top 100 stress at integration points.

Overall, in the high-stress gait cycle, there were also significant differences among the IS and TS groups (*p* < 0.05), as well as among the coupled and uncoupled muscle force groups (*p* < 0.05), as shown in Table 3; Figure 5. The value of *WMV*_*s*−*100*_ of each group was calculated for comparison; the TS group (123.85 MPa) showed higher values than the IS group (114.3 MPa), and the uncoupled muscle force group (125.9 MPa) showed higher values than the coupled muscle force group (119.08 MPa).

**TABLE 3:** Comparison of ${\bar{p}}_{100}$ at the high-stress phase between different groups.

${\bar{p}}_{100}$ at high-stress phases (MPa)	Difference between means	*SE* of the difference	95% *CI* of the difference	*F*	*p*
TS vs. IS group	10.98	3.04	2.533–19.42	13.0	0.022
Uncoupled vs. coupled group	12.84	4.52	0.283–25.40	8.06	0.046

High-stress phases refer to phases 2–4. The average peak stress in the TS group was significantly higher than that in the IS group (*p* < 0.05). The average peak stress in the uncoupled group was significantly higher than that in the coupled group (*p* < 0.05).

IS, iliac screw; TS, transverse screw; uncoupled group, uncoupled muscle force group; coupled group, coupled muscle force group; ${\bar{p}}_{100}$ , mean of the maximum top 100 stress at integration points.

**FIGURE 6:**
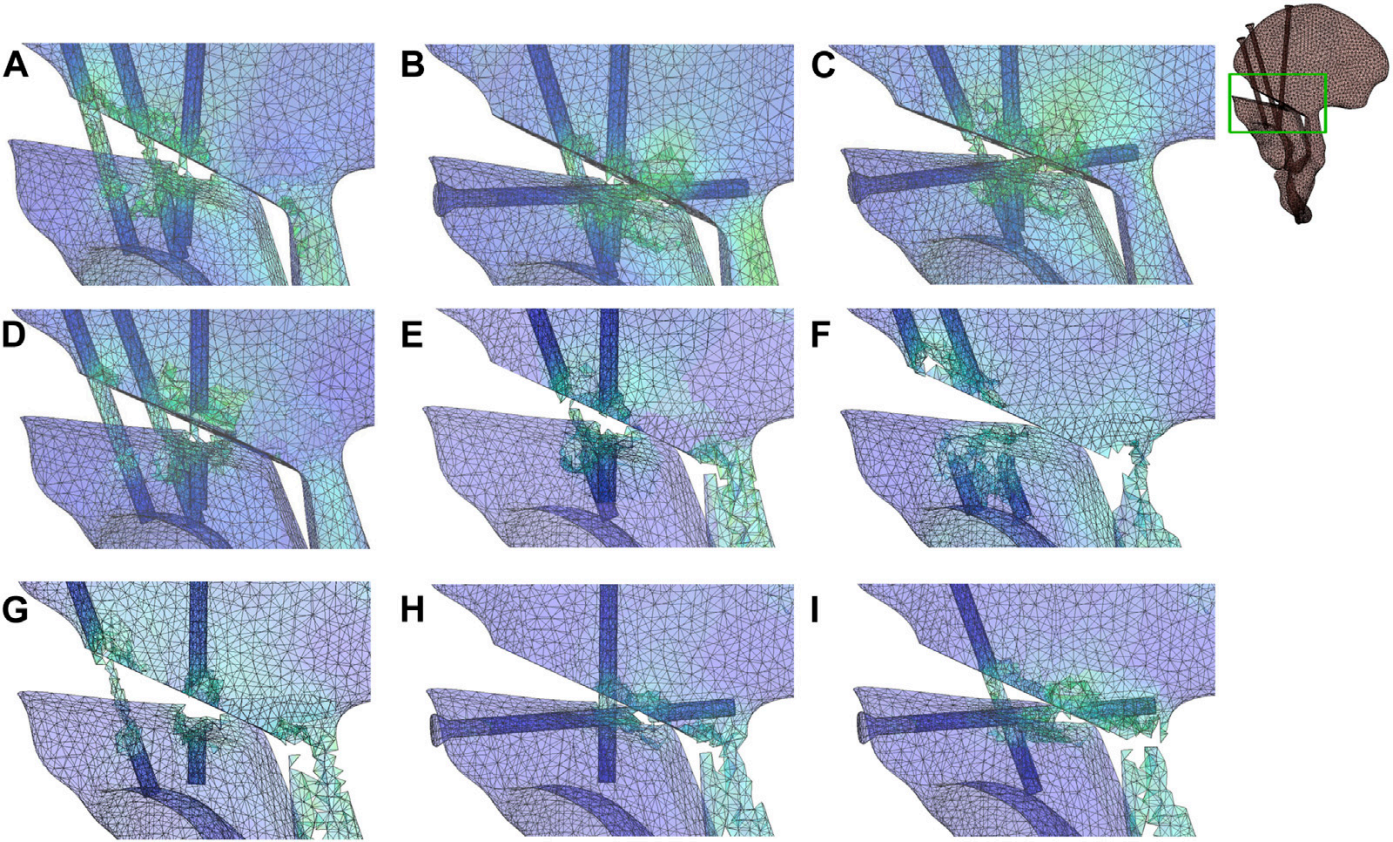
Strain nephograms in different groups at yielding iteration. The *yield* load for the TS group was 6.21*BW **(A)**; the yield load for the IS group was 6.16*BW **(B)**; yield loads for the uncoupled groups were 5.98*BW and 5.81*BW, respectively **(C,D)**; and yield loads for the 2S models were 3.84*BW **(E)**, 3.31*BW **(F)**, 3.70*BW **(G)**, 4.23*BW **(H)**, and 3.64*BW **(I)**. Failed meshes have been deleted. IS, iliac screw; TS, transverse screw; uncoupled group, uncoupled muscle force group; coupled group, coupled muscle force group; the 2S model involved the use of only two screws for fixation.

The failure analysis demonstrated that the number of failed meshes was well below 1% in the models of two-screw internal fixation techniques during the gait cycle. Furthermore, the results of iterations predicted slightly larger yield load for TS configurations (6.21*BW) than the IS (6.16*BW) but well above the gait load (Figures 6A, B). Similar failed mesh distributions were found between the coupled group and uncoupled group but with lower yield load for the uncoupled group (5.9*BW), as shown in Figures 6C,D. The failure analysis of TS and IS (Figures 6A–D) demonstrated the failure status of each model, and simulations predicted the yield load to start at the hole edge of most medial screws, and the fixation failure emerged first at most medial screws, regardless of the group. The failure analysis of the 2S fixation technique revealed lower yield loads and more pronounced bone damage than the IS and TS techniques. As shown in Figures 6E–I, the failure elements were observed near the screws and hole edge as well as extensively involved the posterior column of the acetabulum, indicating severe damage to the posterior column and complete instability of the pelvis. The yield loads for these models were 3.84*BW (Figure 6E), 3.31*BW (Figure 6F), 3.70*BW (Figure 6G), 4.23*BW (Figure 6H), and 3.64*BW (Figure 6I), with the most medial screw absence model (Figure 6F) exhibiting the lowest yield load.

## Discussion

PAO, or periacetabular osteotomy, is a commonly used surgical procedure for preserving the hip joint in the treatment of DDH in young adults and adolescents. During the surgery, intraoperative osteotomy requires internal fixation to maintain stabilization of the acetabular fragment. The IS and TS internal fixation techniques are the mainstream approaches used in PAO, and various clinical and mechanical studies, ranging from *in vitro* experiments to simulations, have been reported (Babis et al., 2002; Yassir et al., 2005; Mechlenburg et al., 2007; Widmer et al., 2010; Bakhtiarinejad et al., 2021; Leopold et al., 2021). However, those mechanical research studies were limited due to the lack of realistic gait simulation, and muscle force that should contribute to the deformation and stress of the bone was not represented in models, leading to underreported FE results (Chen et al., 2019). In this research, we have effectively created and utilized subject-specific postoperative MSK-MBD models for PAO patients using AnyBody technology. We constructed a personalized PAO MSK model by incorporating gait-driven simulation, geometric approximation, and muscle attachment point-enveloping surface optimization. To the best of our knowledge, this is the first time that musculoskeletal modeling techniques have been employed to reveal the mechanical characteristics of postoperative gait behavior in PAO patients, including lower-limb muscle forces and joint reaction forces. The kinematics and IDA output results demonstrated the distinctive characteristics of the hip 6 months after PAO. We observed that there was a significant difference between the preoperative group and healthy group, which means the hip joint remains weak in the early postoperative period.

The mechanical response of whole structures, dominated by HRF, exhibits the classical double peaks during the gait cycle, corresponding to the timing at heel strike and toe-off. This response is primarily attributed to the forces generated by the internal muscle contractions and the external ground reaction force. These factors play a crucial role in the efficient execution of human locomotion (Correa et al., 2010). HRFs in the PAO group presented lower overall trends, which were characterized, as the second peaks were lower and flatter and even disappeared. The second peaks mainly benefited from muscle contractions, which were dominated by abductor muscles (Ebert et al., 2017; Lanza et al., 2021) (mean maximal value over 1.2*BW), mainly including gluteus minor, gluteus medius, and gluteus maximus superior parts. During the second peak phases, the related muscles forcefully contracted to match the lower extremity, leaving the ground and maintaining the balance of the pelvis and body weight center. DDH patients commonly present with weakened or insufficient strength in abductor muscles, which leads to noticeable irregularities in their gait or stance. These irregularities are recognized as significant clinical manifestations of DDH (Liu et al., 2012; Loverro et al., 2019; Song et al., 2020). The results of IDA have revealed this phenomenon and shown significant improvement from the preoperative to early postoperative period. However, it should be noted that although PAO surgery can restore the DDH to the normal anatomical structure, these transformations will take time. A literature reported almost similar results that peri-ilium muscle strength could be improved but not normalized in 1 year post-PAO (Gahramanov et al., 2017). Lower-joint mobility and muscle strength were more pronounced in abduction and rotation and therefore recommended for enhancing appropriate coordination and stability.

Muscle is considered to be attached to the surface of the bone, and the muscle force can contribute to the deformation and stress of the bone during muscle contraction and stretching; therefore, the difference in muscle insertion and the working structure will affect the bone mechanics. In this study, we used a morphological scaling algorithm to modify the original geometry shape of the pelvis in the general musculoskeletal model to match the individual pelvis bone morphology based on a CT data reconstruction model to obtain more accurate peri-ilium muscle insertion and perform more realistic simulation (Phillips et al., 2007). Furthermore, the IDA output results were coupled with the FE model for mechanical analysis.

This study highlights the crucial influence of muscle boundary conditions on the accuracy of FEA. Therefore, it is essential to implement the aforementioned settings for more precise analysis. The stress maps depict light blue regions that indicate moderate stress, primarily concentrated on the posterior column of the acetabulum in coupled models. However, a lower ${\bar{p}}_{100}$ value signifies lower peak stress across the entire structure. Importantly, the coupled group demonstrated a lower concentration of stress around the screws, indicating that muscle forces may counteract the hip reaction force and play a protective role in maintaining stability. Although the hip reaction force may decrease in early postoperative gait, weaker muscle strength may compromise the overall mechanostability (Freke et al., 2018). The results of the FEA indicated that the TS group exhibited a more obvious concentration of stress, as well as greater mean peak stress, during the gait, especially at high-stress phases. Stress nephograms indicated that the stress concentration areas mainly surrounded the osteotomy site above the acetabular fragment, corresponding to the lower-middle segments of screws and the nail holes of bone, which were similarly more pronounced at the most medial screws in both IS and TS fixation groups. The failure simulation results also revealed that the most medial screws in both fixation techniques experienced localized element failure first. This indicates that the most medial screws have a significant impact on the overall pelvic stability and fixation strength after PAO. Therefore, surgeons should carefully consider how to ensure better accurate implantation (Jiang et al., 2022). Furthermore, the failure analysis demonstrated that two-screw internal fixation techniques could provide sufficient capacity for the strain resistance to failure during the early PAO postoperative gait even in high-stress phases, although the TS group showed higher yield load, which is well higher than the gait load. Similar results have been reported by Babis et al. (2002) in an *in vitro* mechanics test on fresh cadaver specimens, where the TS technique provided significantly greater local stiffness to the construct fixation and higher ultimate loads beyond which catastrophic failure occurs. However, the *in vitro* mechanics test is not valid at simulating the mechanical conditions during daily activities, as well as the complex corresponding muscle responses (Mo et al., 2020; Putra et al., 2022). The failure analysis of the 2S fixation technique demonstrates lower yield loads and more pronounced bone damage than that of the IS and TS techniques. This indicates that the use of only two screws for fixation does not provide sufficient stability for the pelvis. Three screws offer better mechanical advantages. The 2S fixation technique model with the most medial screw absence exhibits the lowest yield load, further confirming the significant impact of the most medial screw on the pelvic stability and fixation strength after PAO surgery. From a comprehensive standpoint, we maintain that the TS technique does not show promising superiority or advantages in terms of biomechanical performance compared to the IS technique. As such, it is crucial to focus on improving the placement of the most medial screw, which plays a pivotal role in stress conduction and may potentially contribute to better outcomes for patients undergoing PAO.

However, limitations are noteworthy. Similar to the study by Ko et al. (2002), simplified settings in the FE models were applied; for example, internal fixation screws were geometrically simulated as smooth cylindrical solids without the thread design to obtain feasible running and improved solver stability. Additionally, the inclusion of only 12 subjects may have resulted in a sample size that was too small to accurately depict reality. It is worth noting that our study was designed to include patients who underwent PAO for unilateral DDH. We controlled for imaging classification and physical fitness to ensure consistency in baseline data among those who were included, which led to the exclusion of some patients who did not meet the criteria. Nevertheless, the objective of this study is to introduce a new co-simulation framework that can produce more realistic simulations of PAO by incorporating specific postoperative gait patterns and skeletal morphological parameters. Ongoing enhancements are necessary for this co-simulation framework, such as taking into account the impact of variations in implant placement, reorientation of the acetabular fragment, and patient demographics.

## Conclusion

In this study, we presented a detailed procedure for investigating the biomechanics of the pelvis and implants after PAO, which could assist the surgeon in better assessing the surgery. Our results confirmed that lower-limb mobility, HRF, and peri-ilium muscle strength occur in the early postoperative course. Moreover, TS and IS internal fixation techniques can provide sufficient capacity for strain resistance to failure during early PAO postoperative gait. Compared to the IS technique, the TS technique showed higher average peak stress, particularly during the second–fourth high-stress phases. This suggests a more significant stress concentration in TS, which is considered to pose a higher risk of cyclic fatigue failure during daily gait. Therefore, we do not support the notion that the TS technique demonstrates promising superiority or advantages in biomechanical performance over the IS technique during the early postoperative gait after PAO. It is worth noting that both the TS and IS techniques exhibited maximum stress concentration and element failure in the most medial screws, indicating their significant impact on the overall pelvic stability and fixation strength following PAO.

## Data Availability

The original contributions presented in the study are included in the article/Supplementary Material; further inquiries can be directed to the corresponding authors.
