# Supplementary figures and images for: Mechanical upside of PAO mainstream fixations: co-simulation based on early postoperative gait characteristics of DDH patients
Source: Front Bioeng Biotechnol. 2023 Jul 19;11:1171040. doi: 10.3389/fbioe.2023.1171040 (PMC10396769; doi:10.3389/fbioe.2023.1171040)

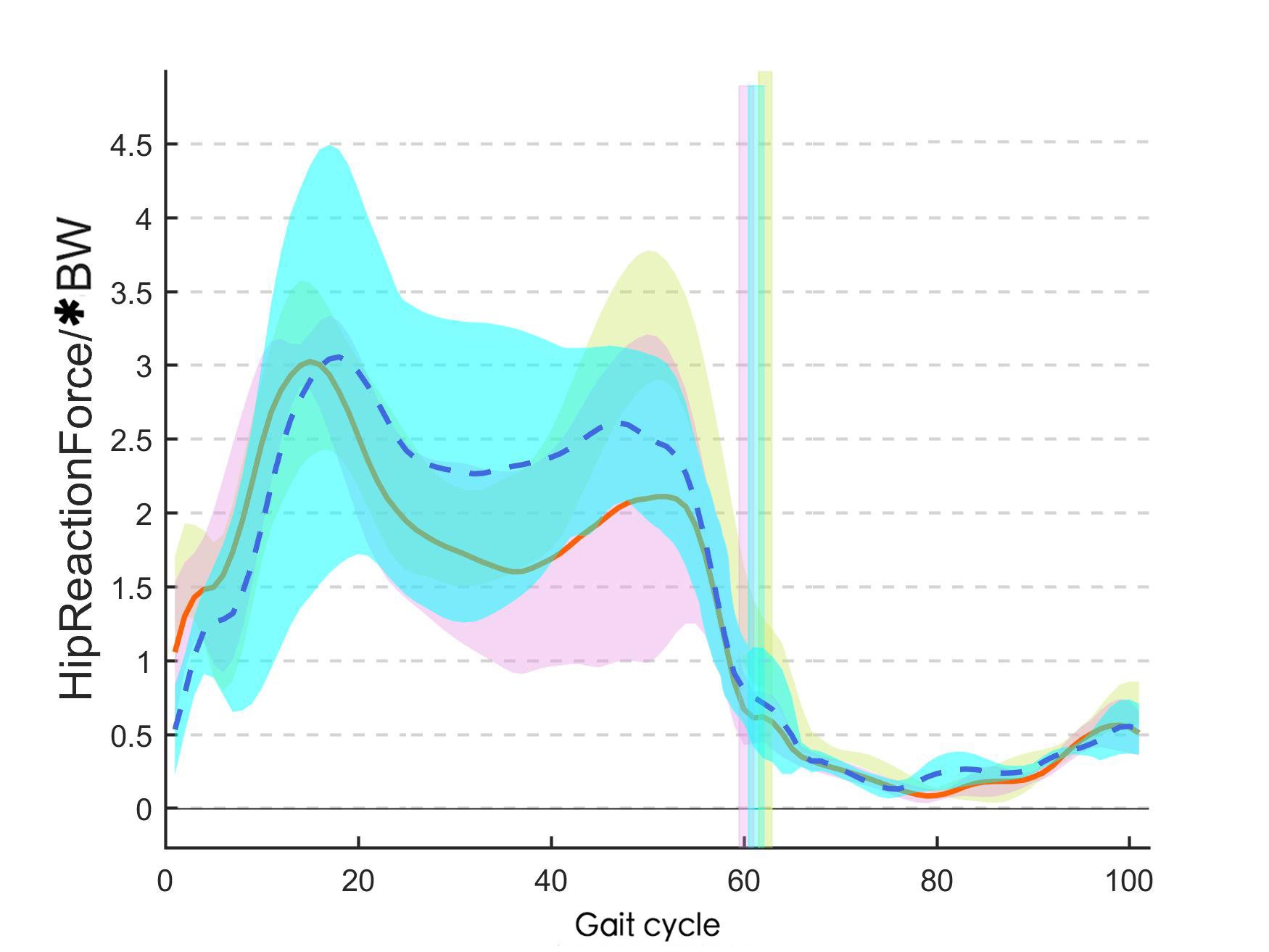

Supplement: Supplementary file 1 [file DataSheet1.ZIP › IDA RESULTS/dl2.jpg]

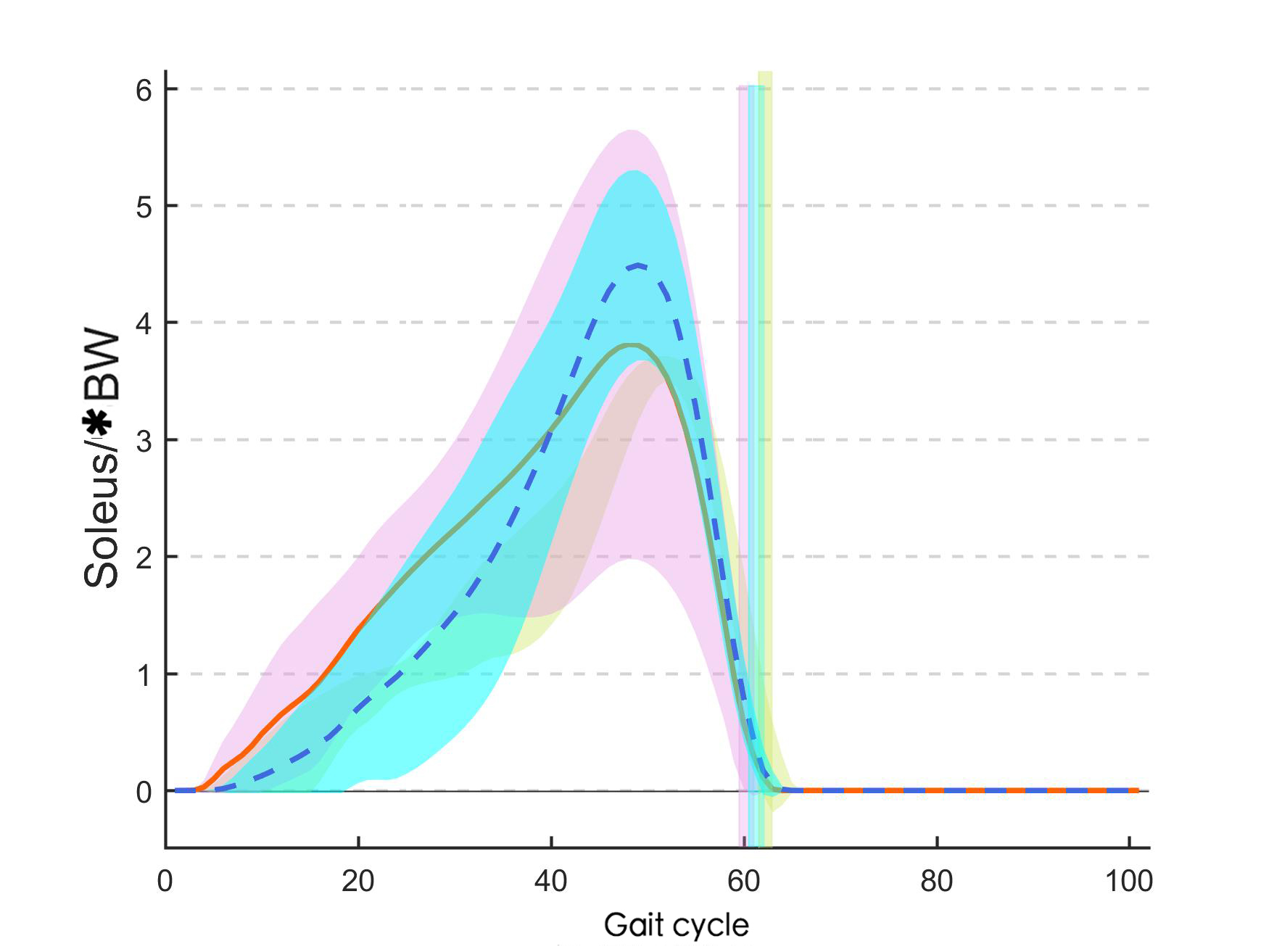

Supplement: Supplementary file 1 [file DataSheet1.ZIP › IDA RESULTS/dl40.jpg]

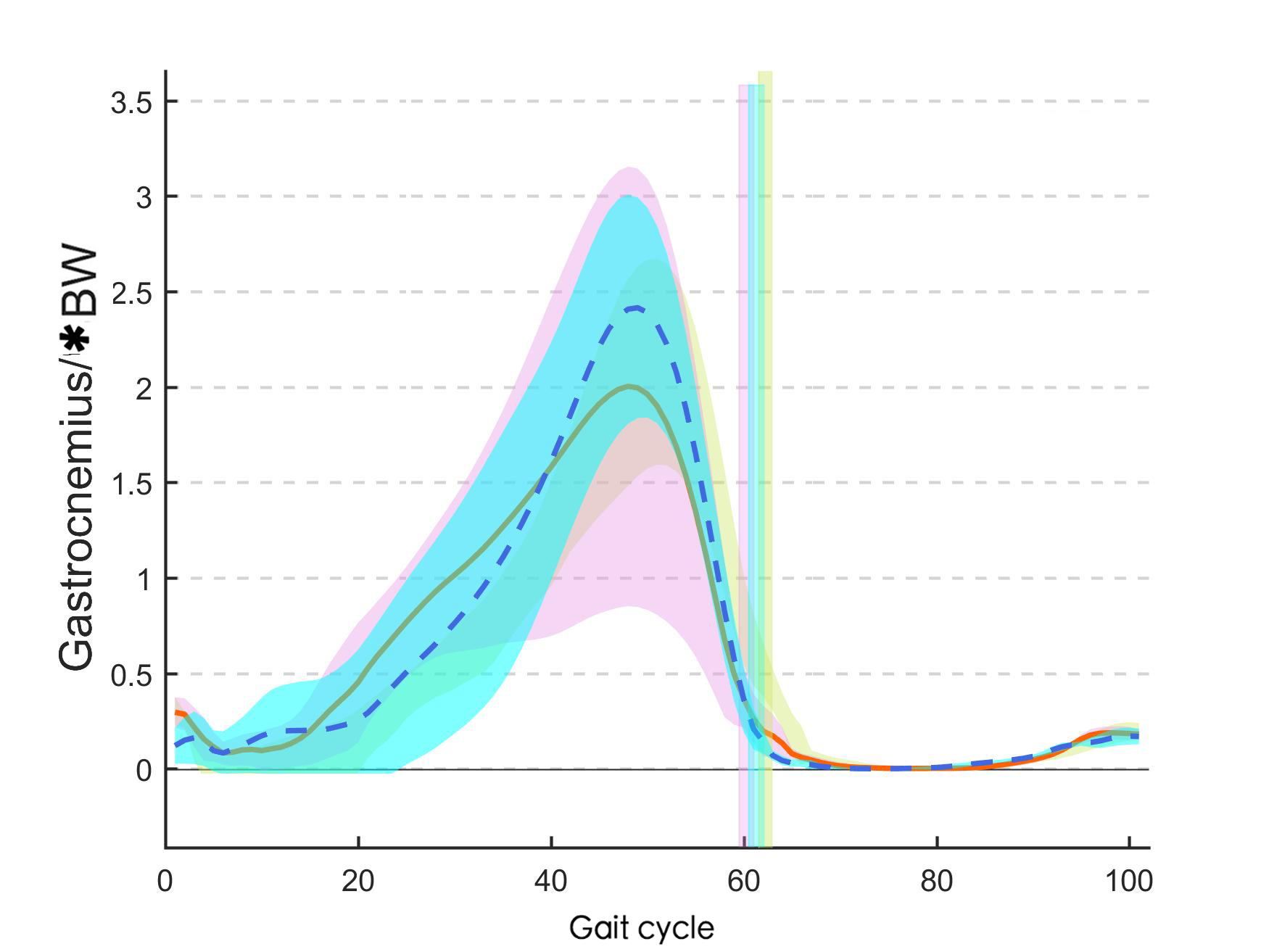

Supplement: Supplementary file 1 [file DataSheet1.ZIP › IDA RESULTS/dl41.jpg]

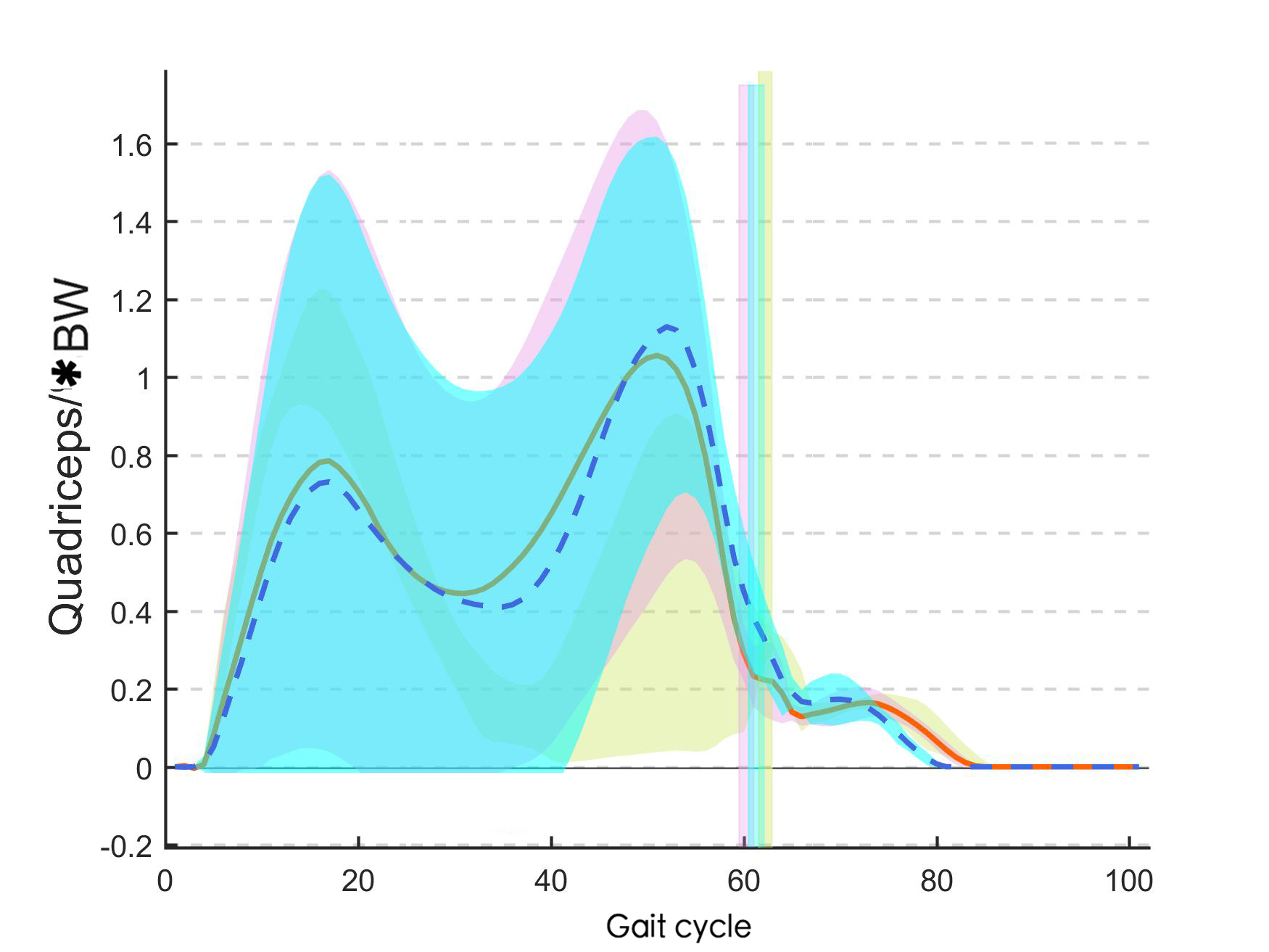

Supplement: Supplementary file 1 [file DataSheet1.ZIP › IDA RESULTS/dl42.jpg]

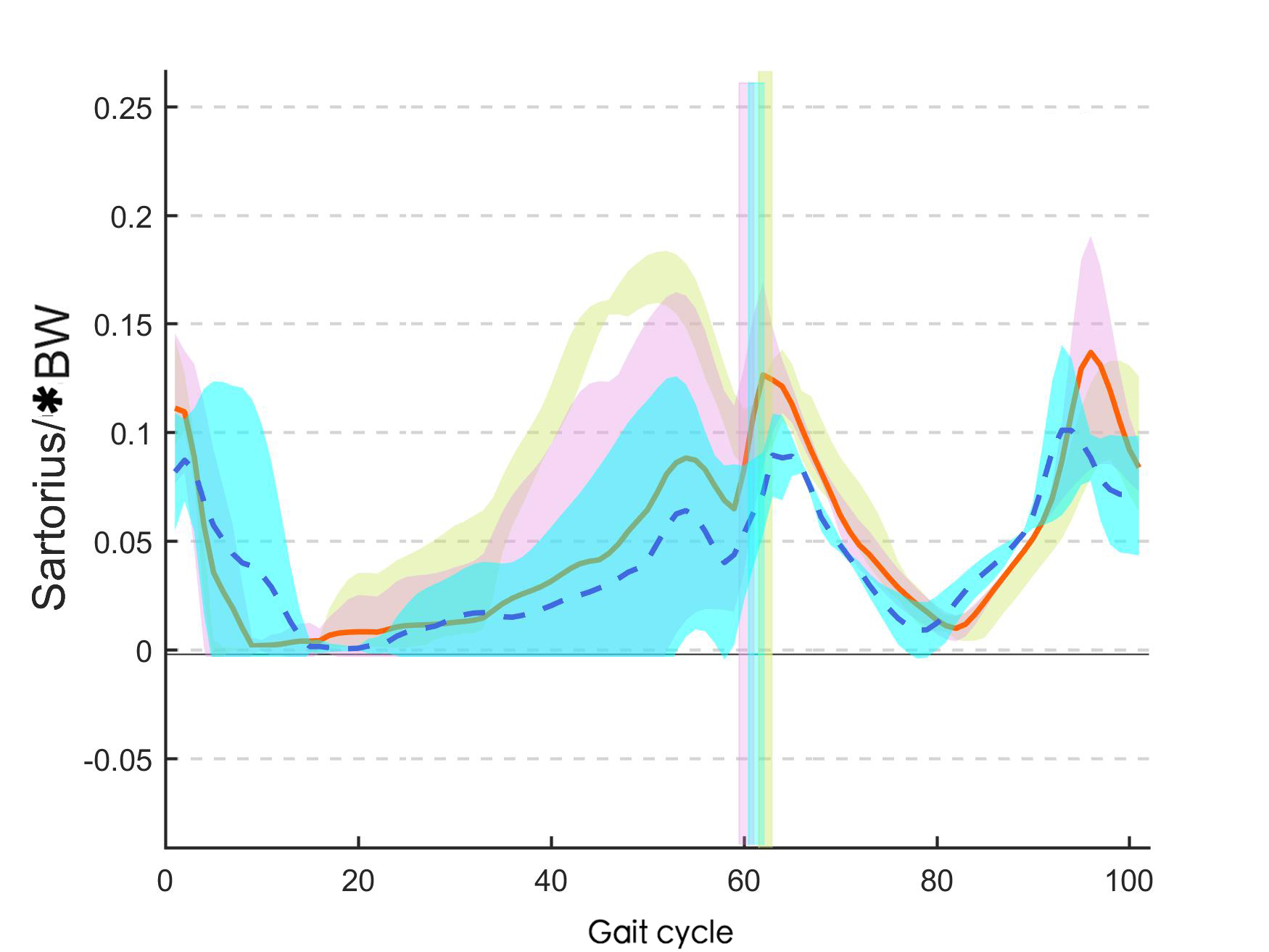

Supplement: Supplementary file 1 [file DataSheet1.ZIP › IDA RESULTS/dl43.jpg]

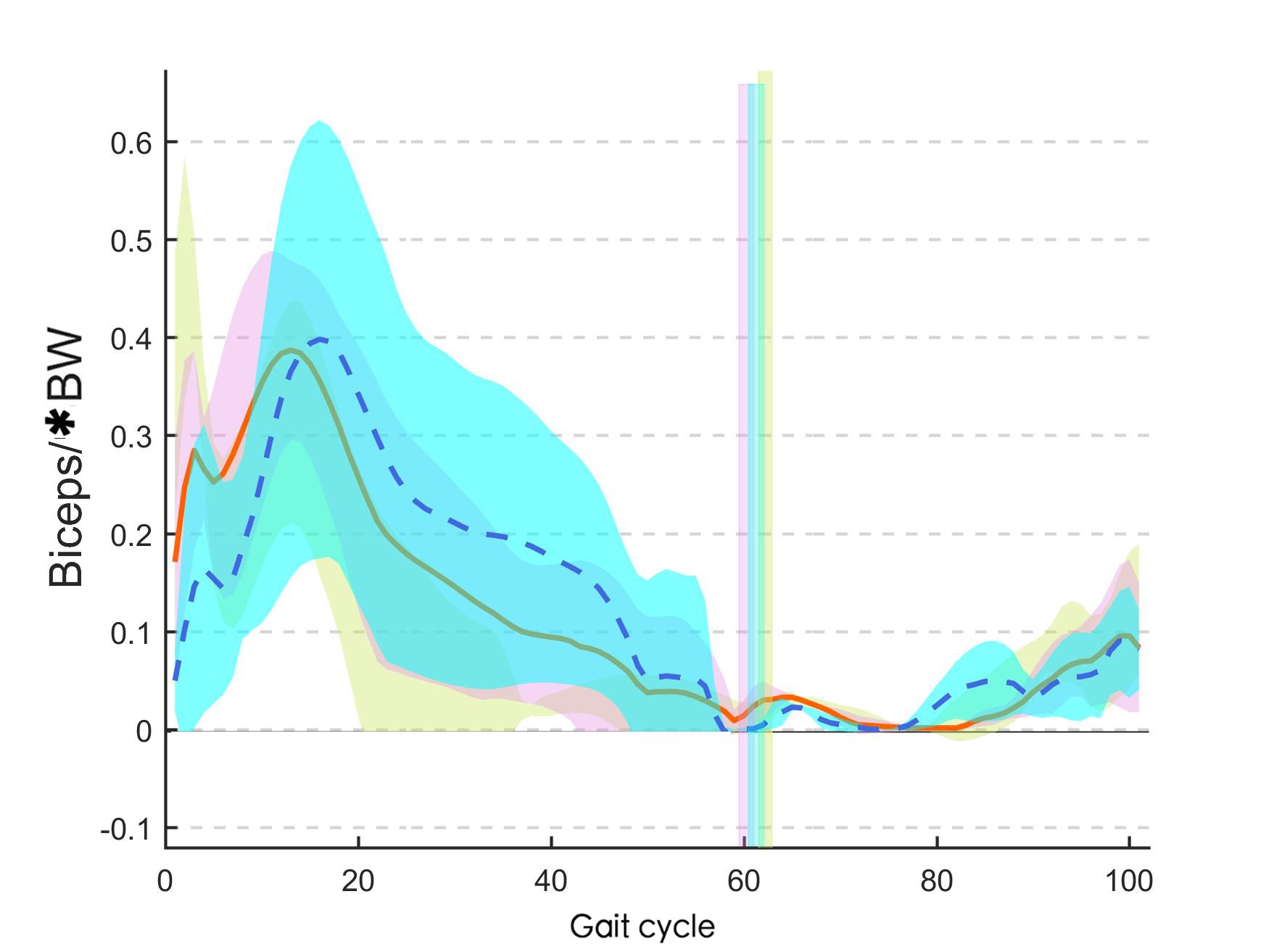

Supplement: Supplementary file 1 [file DataSheet1.ZIP › IDA RESULTS/dl44.jpg]

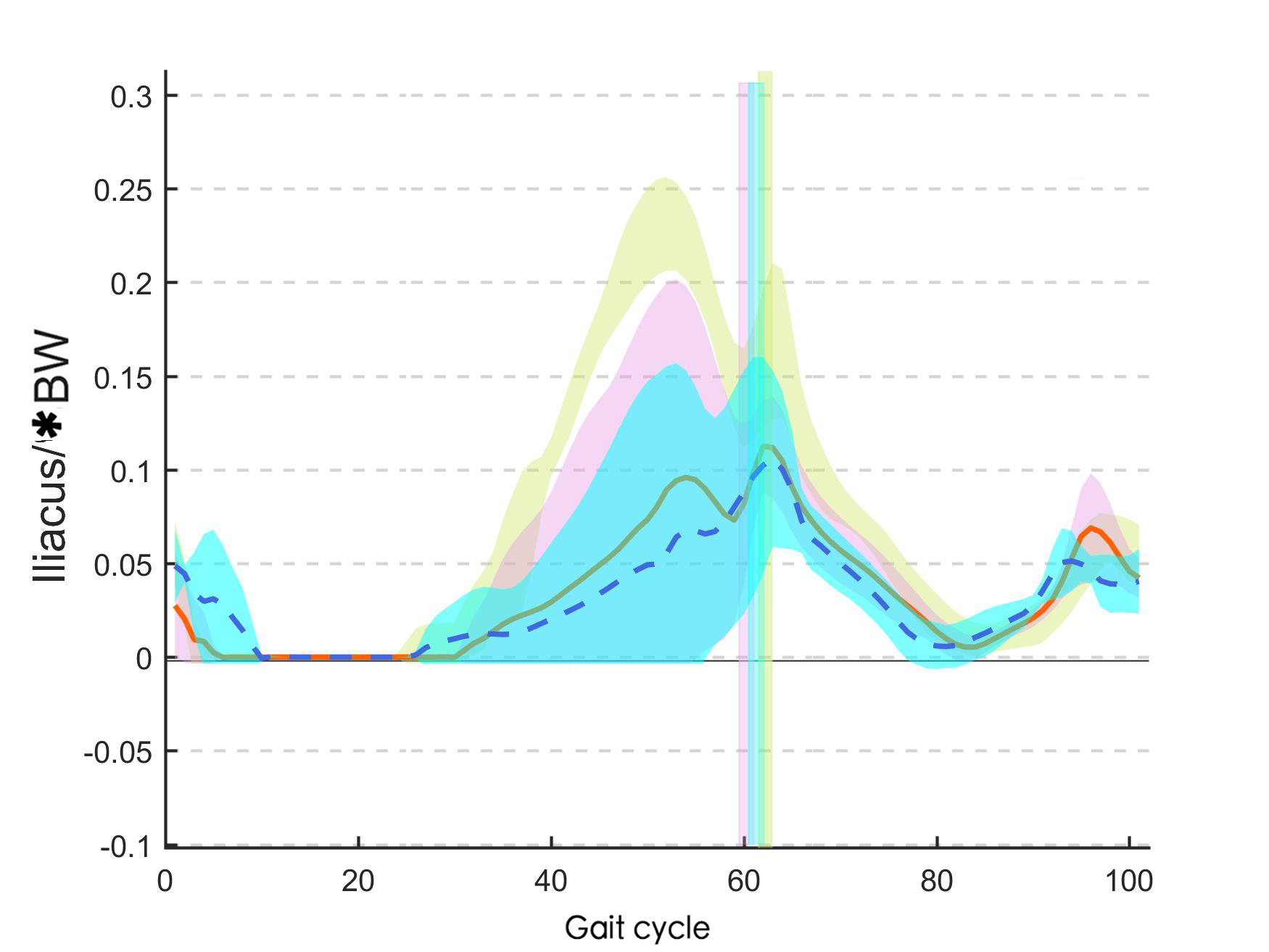

Supplement: Supplementary file 1 [file DataSheet1.ZIP › IDA RESULTS/dl45.jpg]

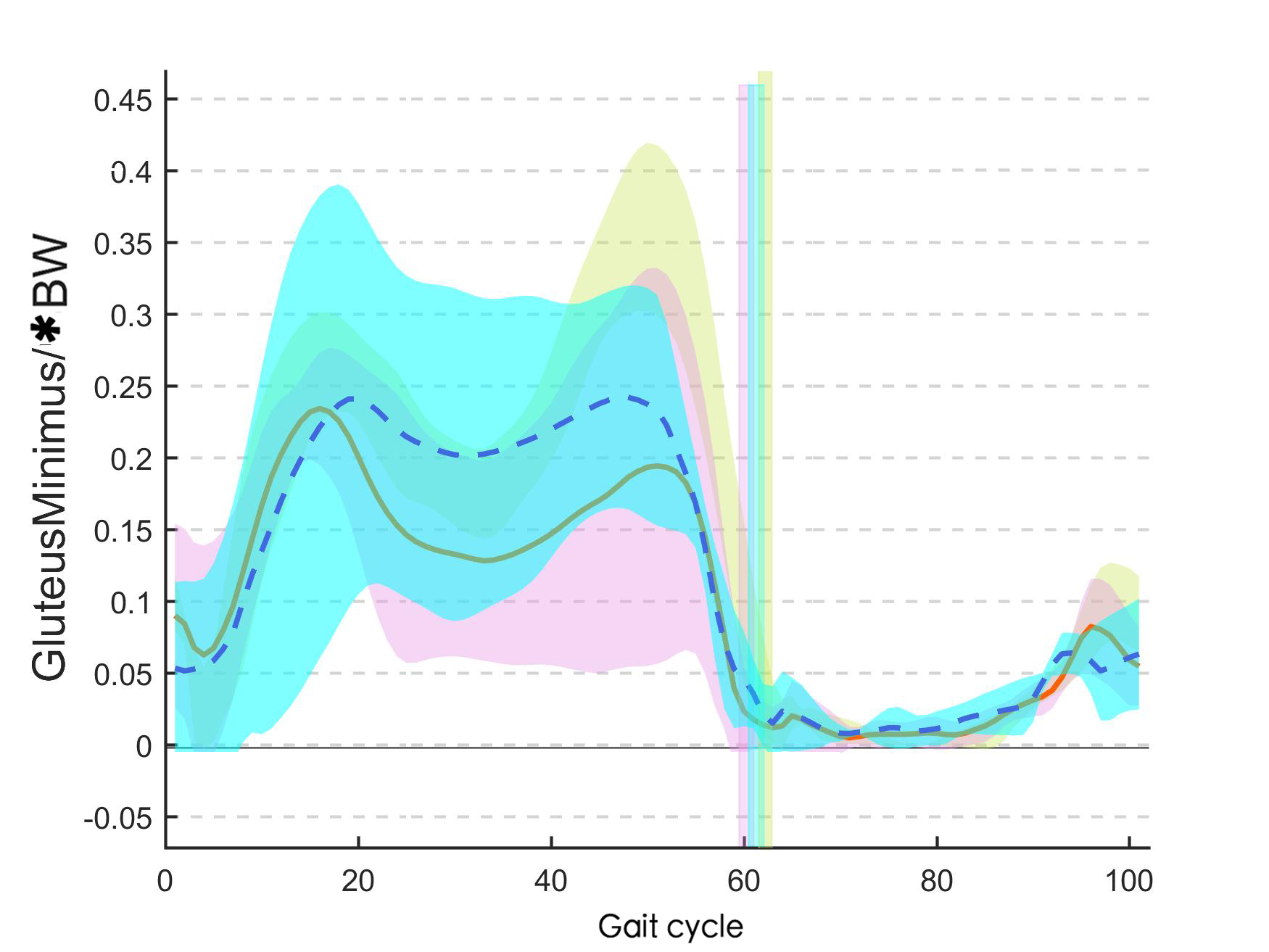

Supplement: Supplementary file 1 [file DataSheet1.ZIP › IDA RESULTS/dl46.jpg]

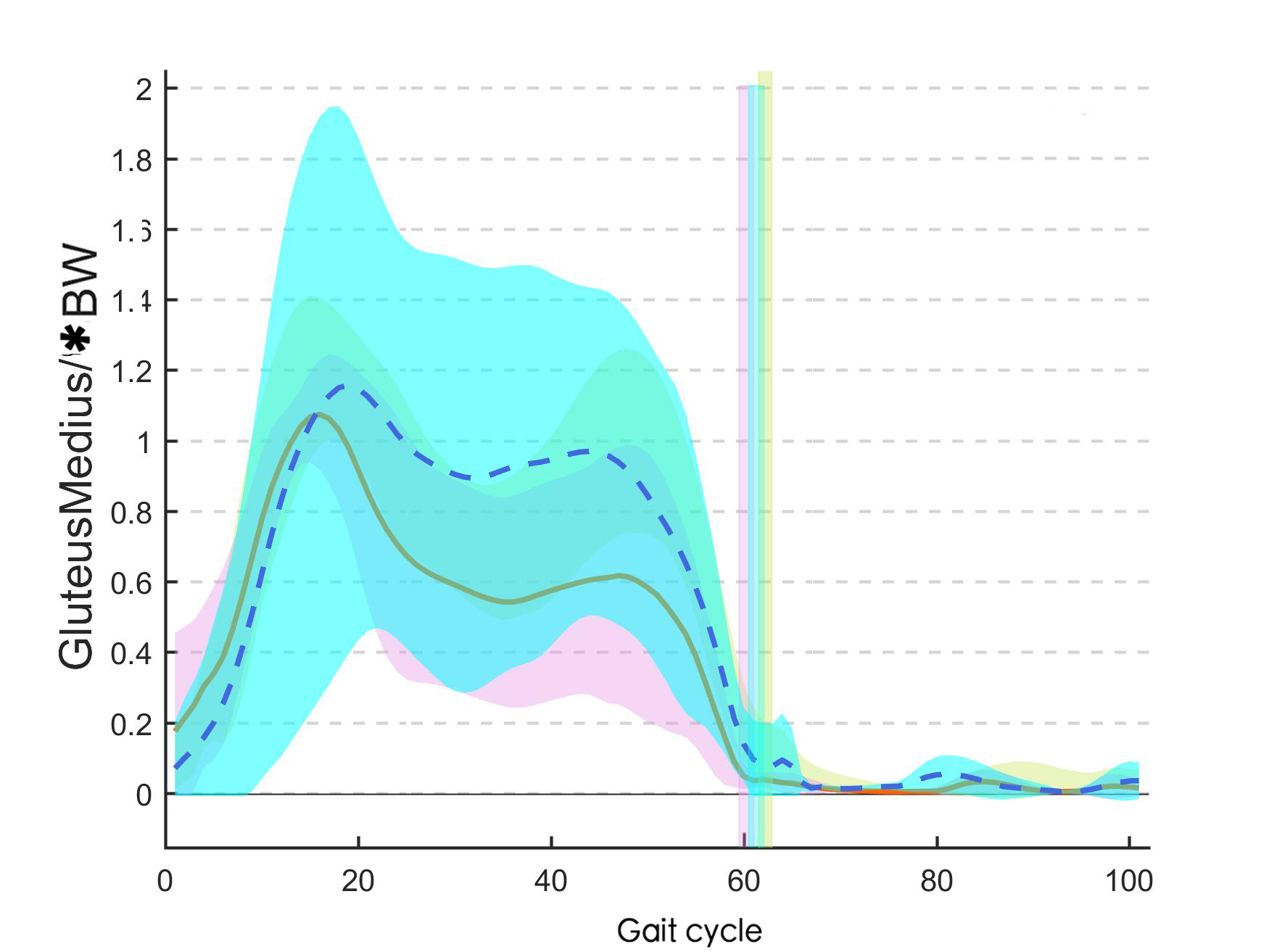

Supplement: Supplementary file 1 [file DataSheet1.ZIP › IDA RESULTS/dl47.jpg]

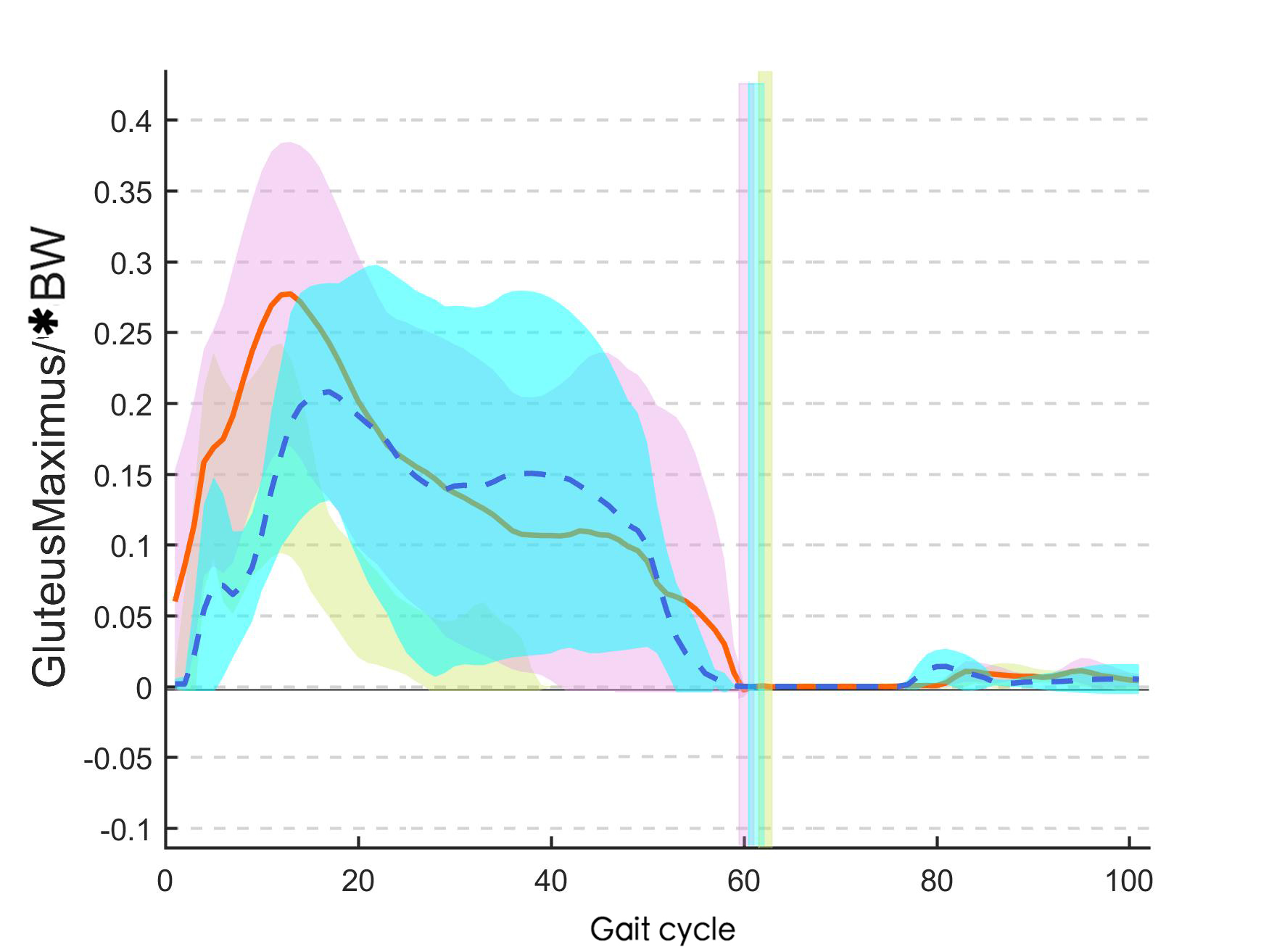

Supplement: Supplementary file 1 [file DataSheet1.ZIP › IDA RESULTS/dl48.jpg]

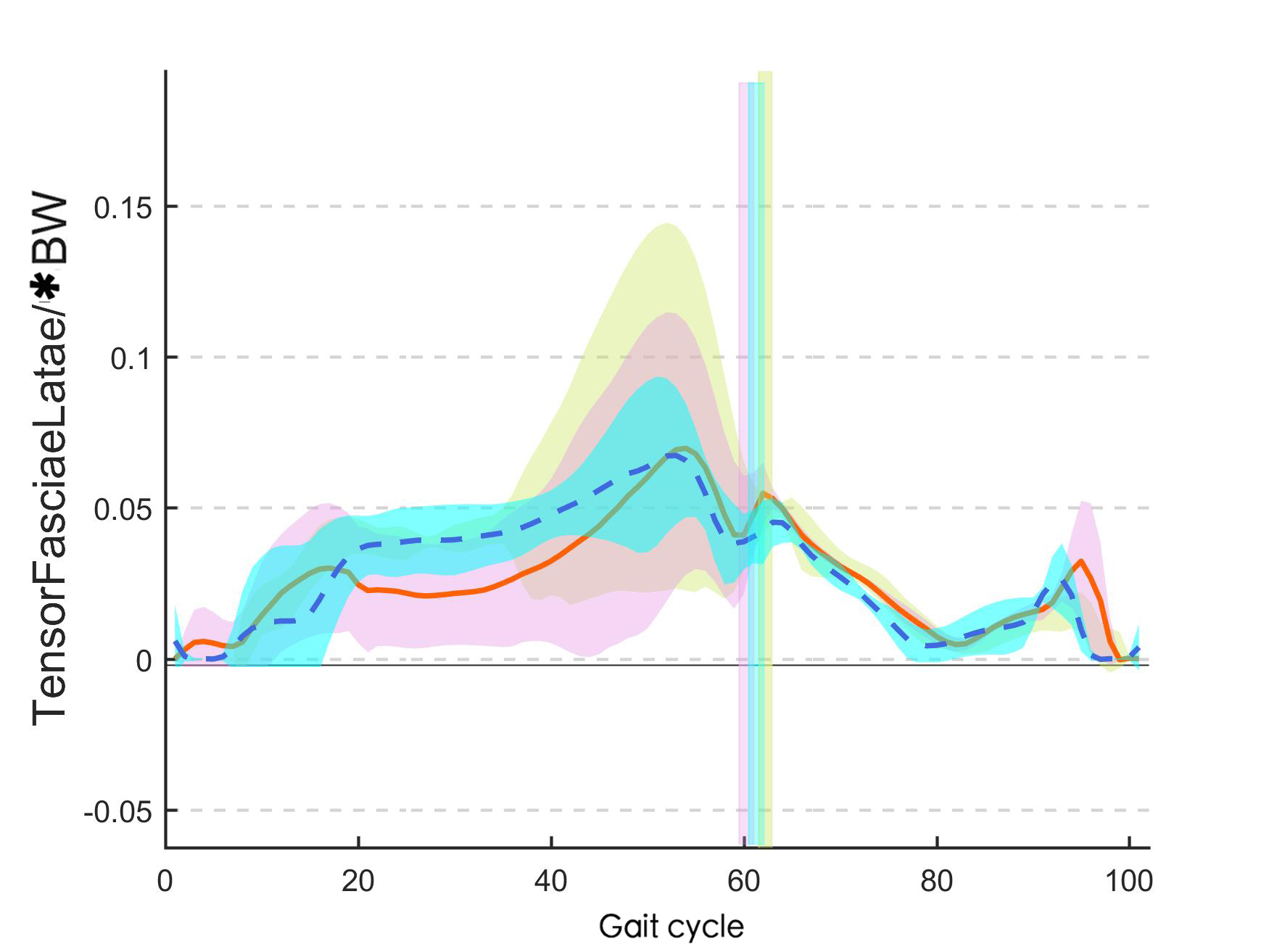

Supplement: Supplementary file 1 [file DataSheet1.ZIP › IDA RESULTS/dl49.jpg]

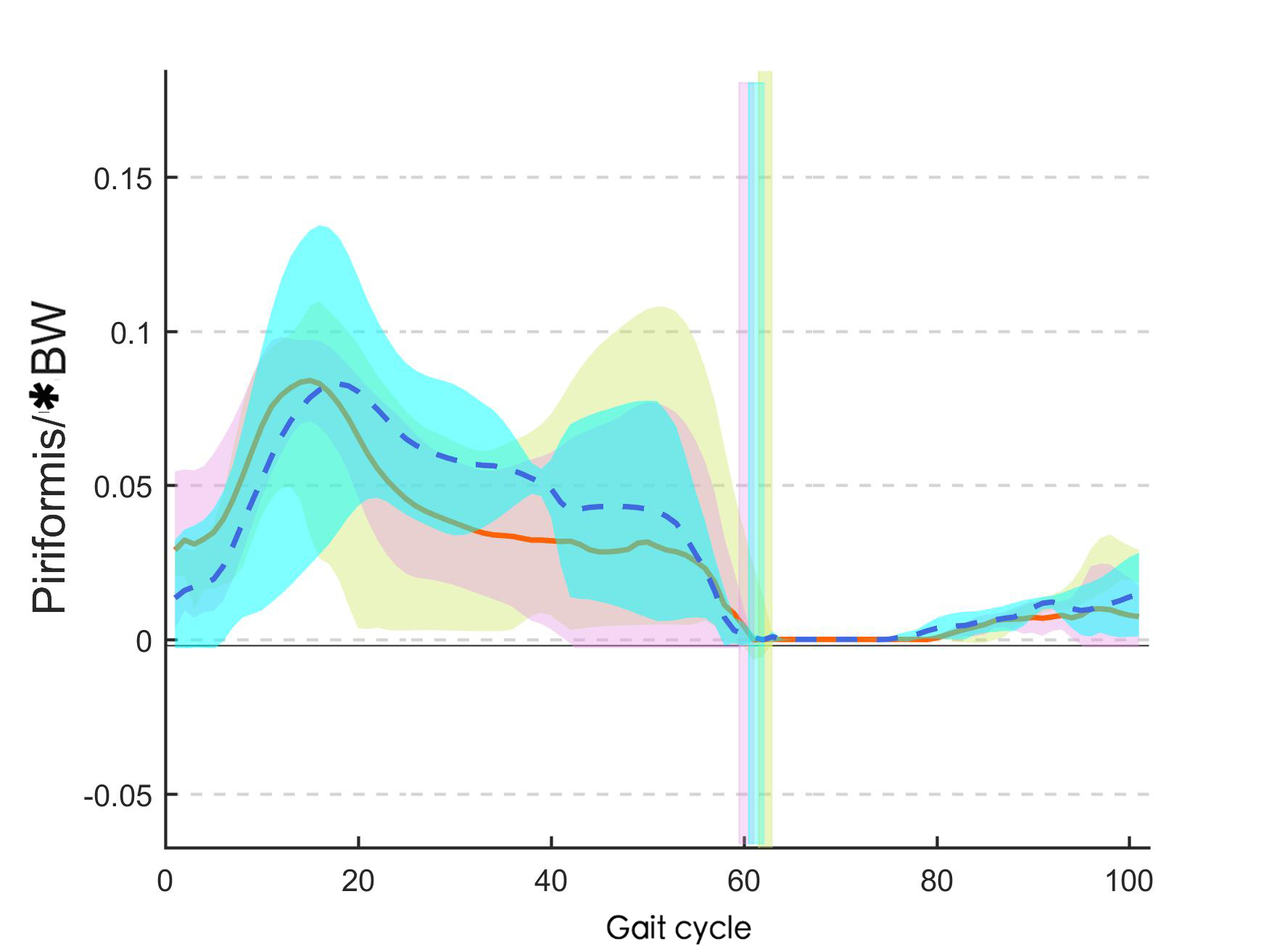

Supplement: Supplementary file 1 [file DataSheet1.ZIP › IDA RESULTS/dl50.jpg]

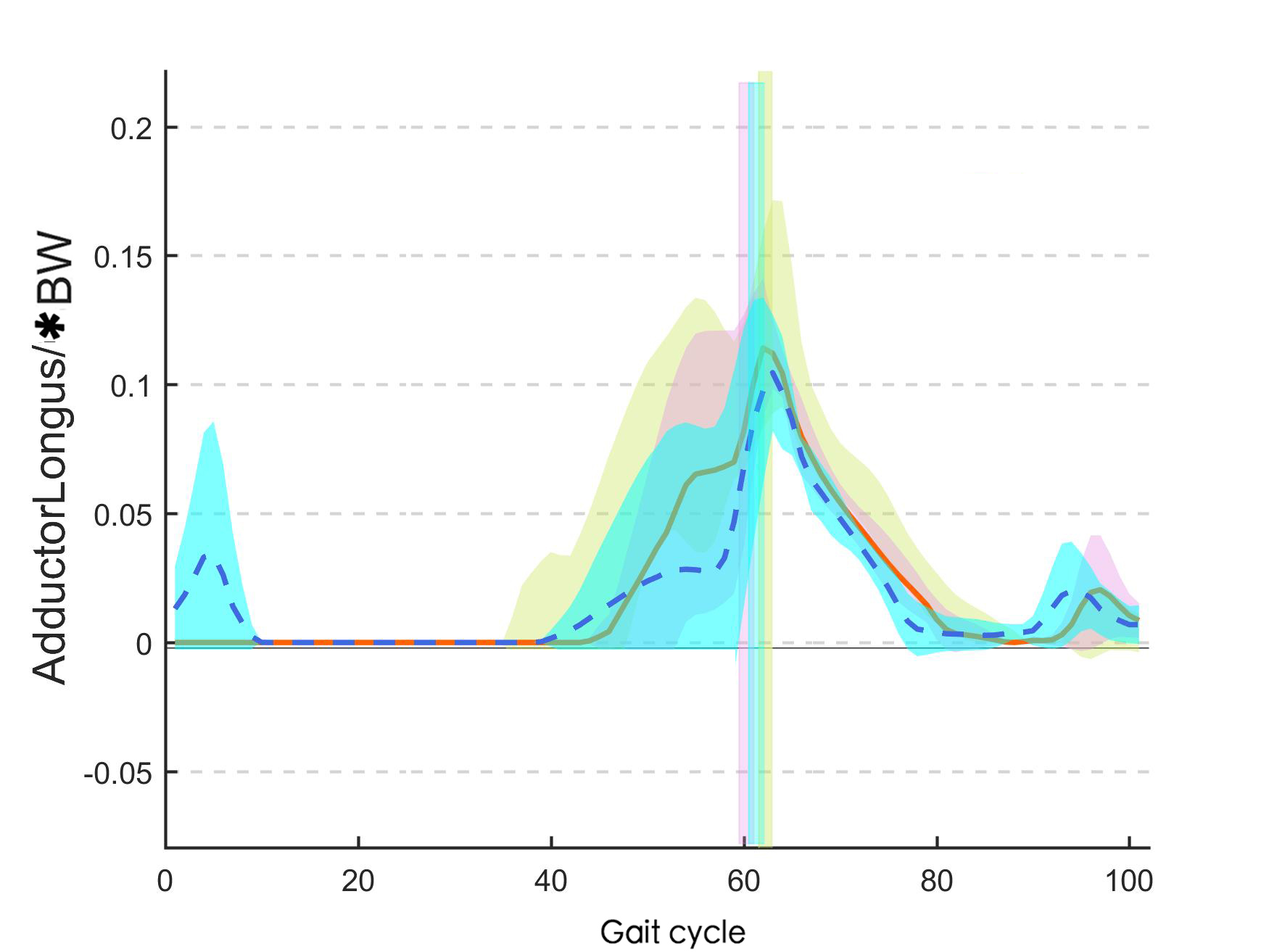

Supplement: Supplementary file 1 [file DataSheet1.ZIP › IDA RESULTS/dl51.jpg]

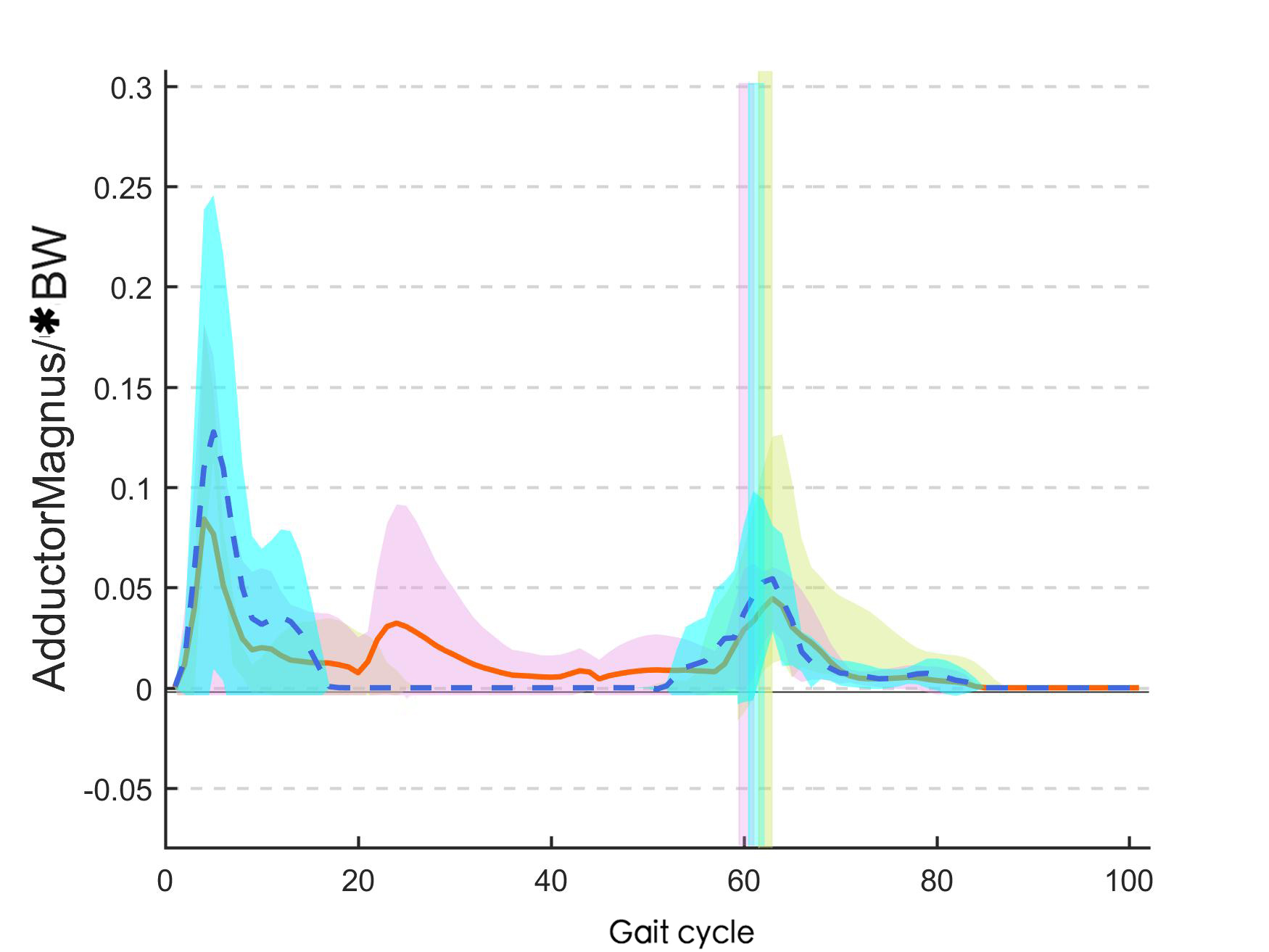

Supplement: Supplementary file 1 [file DataSheet1.ZIP › IDA RESULTS/dl52.jpg]

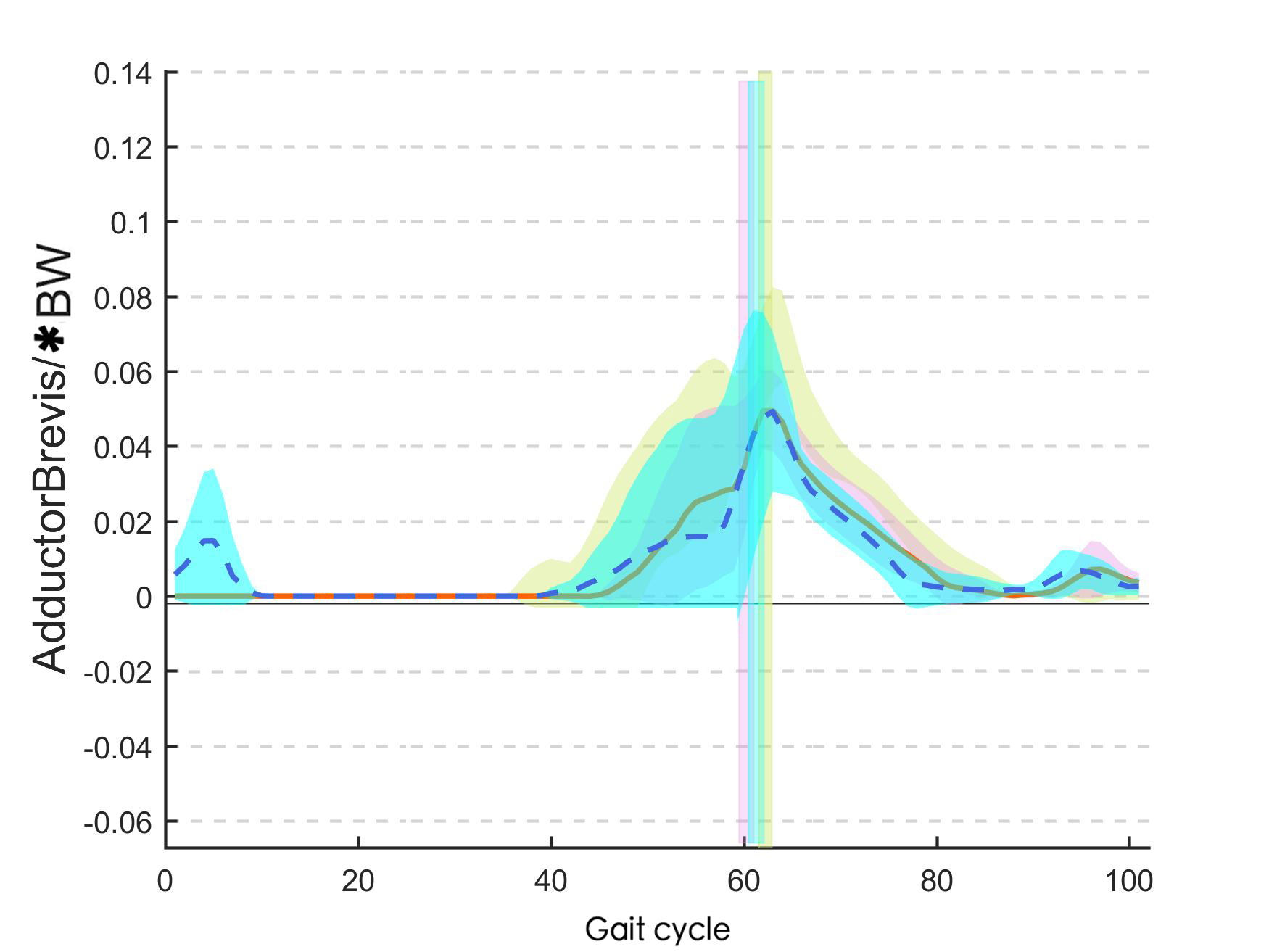

Supplement: Supplementary file 1 [file DataSheet1.ZIP › IDA RESULTS/dl53.jpg]

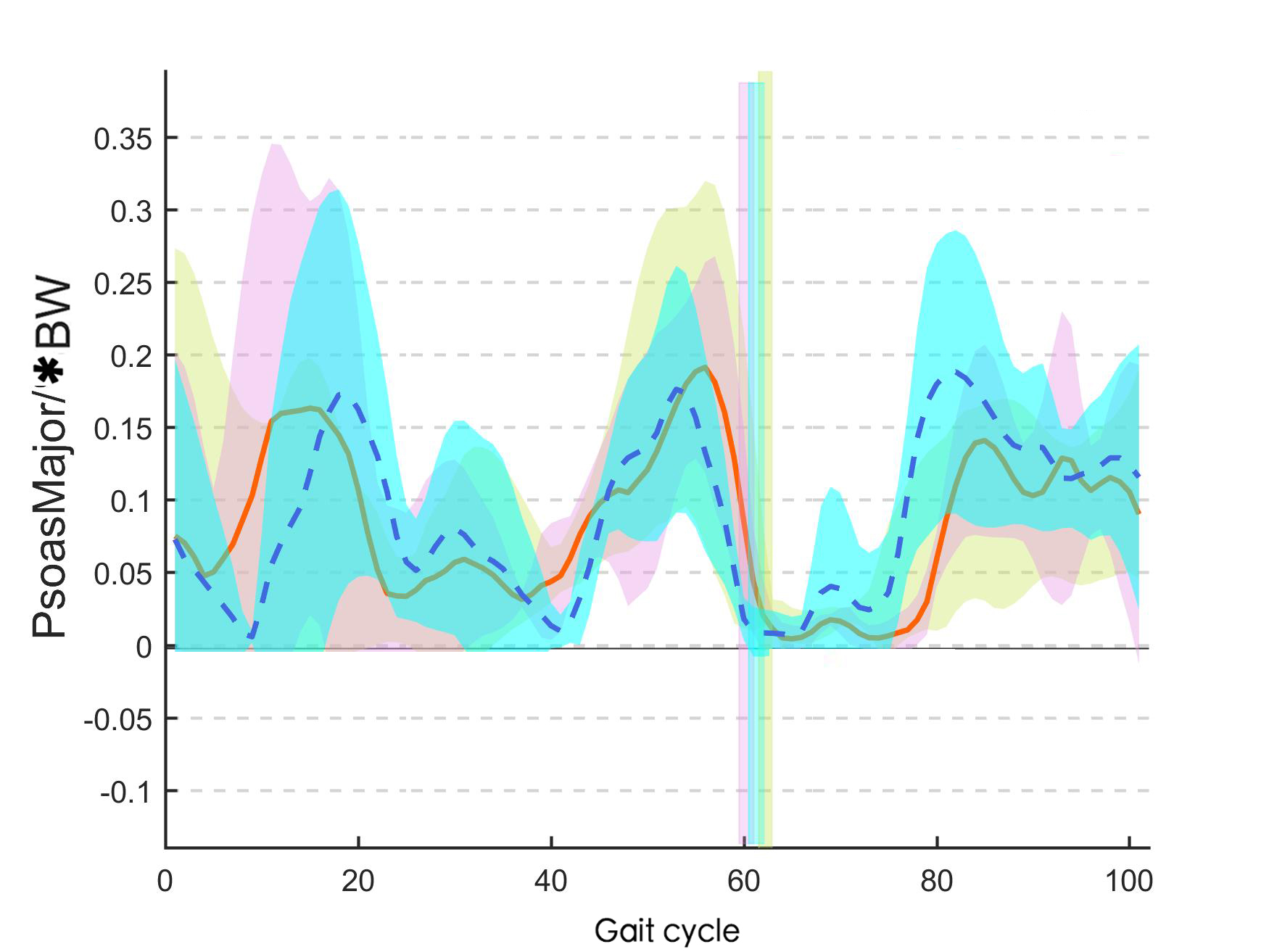

Supplement: Supplementary file 1 [file DataSheet1.ZIP › IDA RESULTS/dl54.jpg]

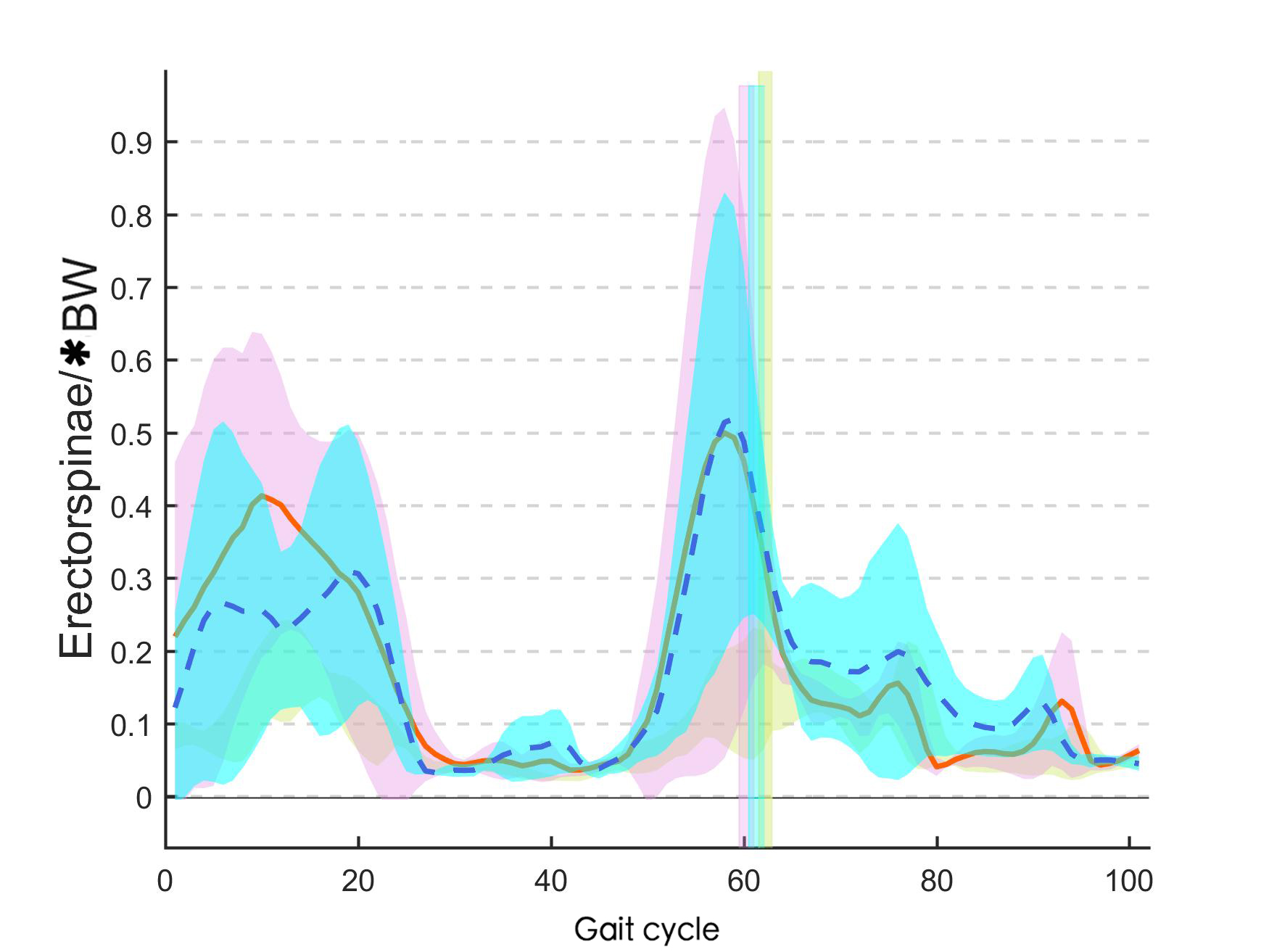

Supplement: Supplementary file 1 [file DataSheet1.ZIP › IDA RESULTS/dl55.jpg]

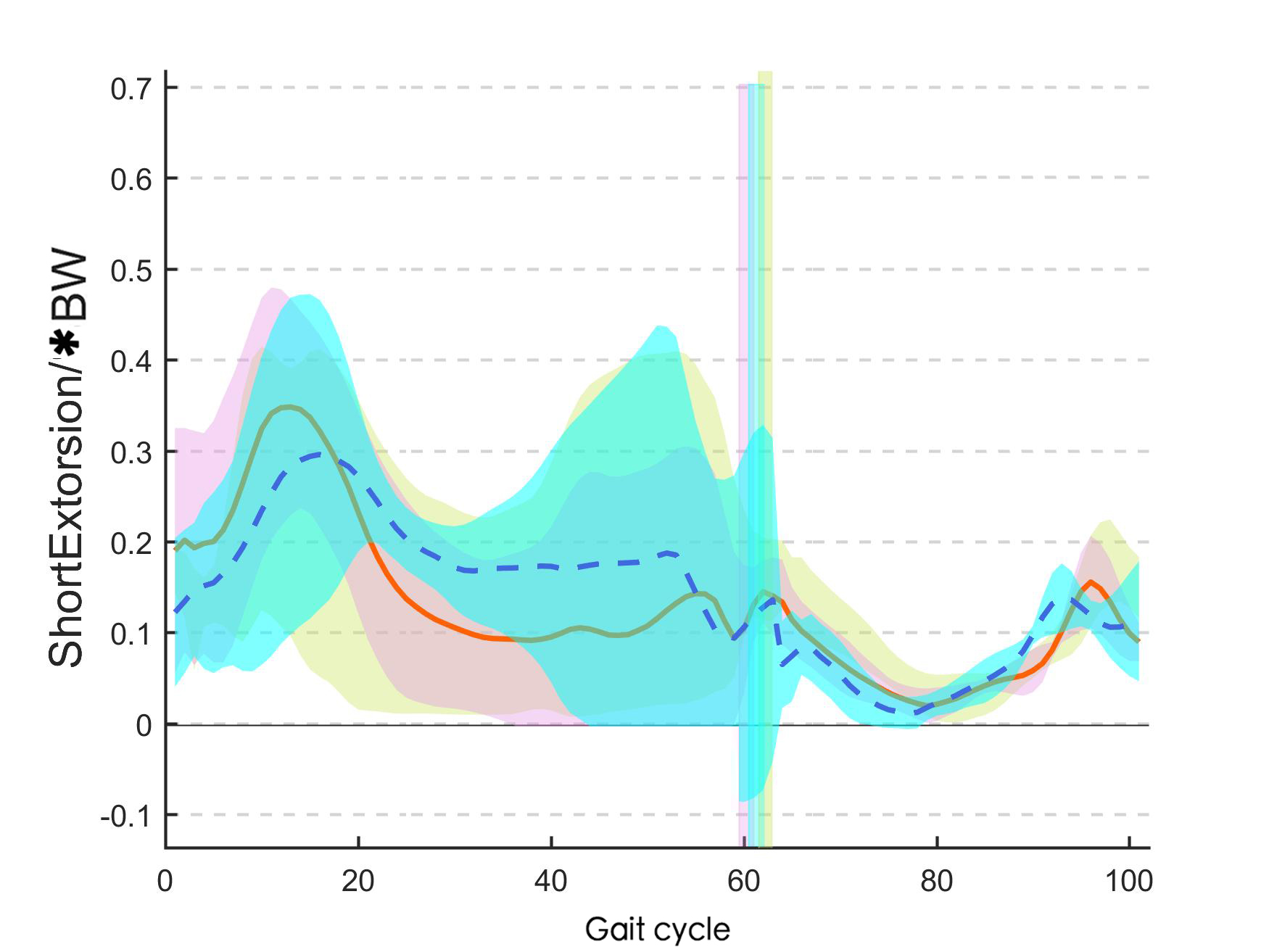

Supplement: Supplementary file 1 [file DataSheet1.ZIP › IDA RESULTS/dl56.jpg]

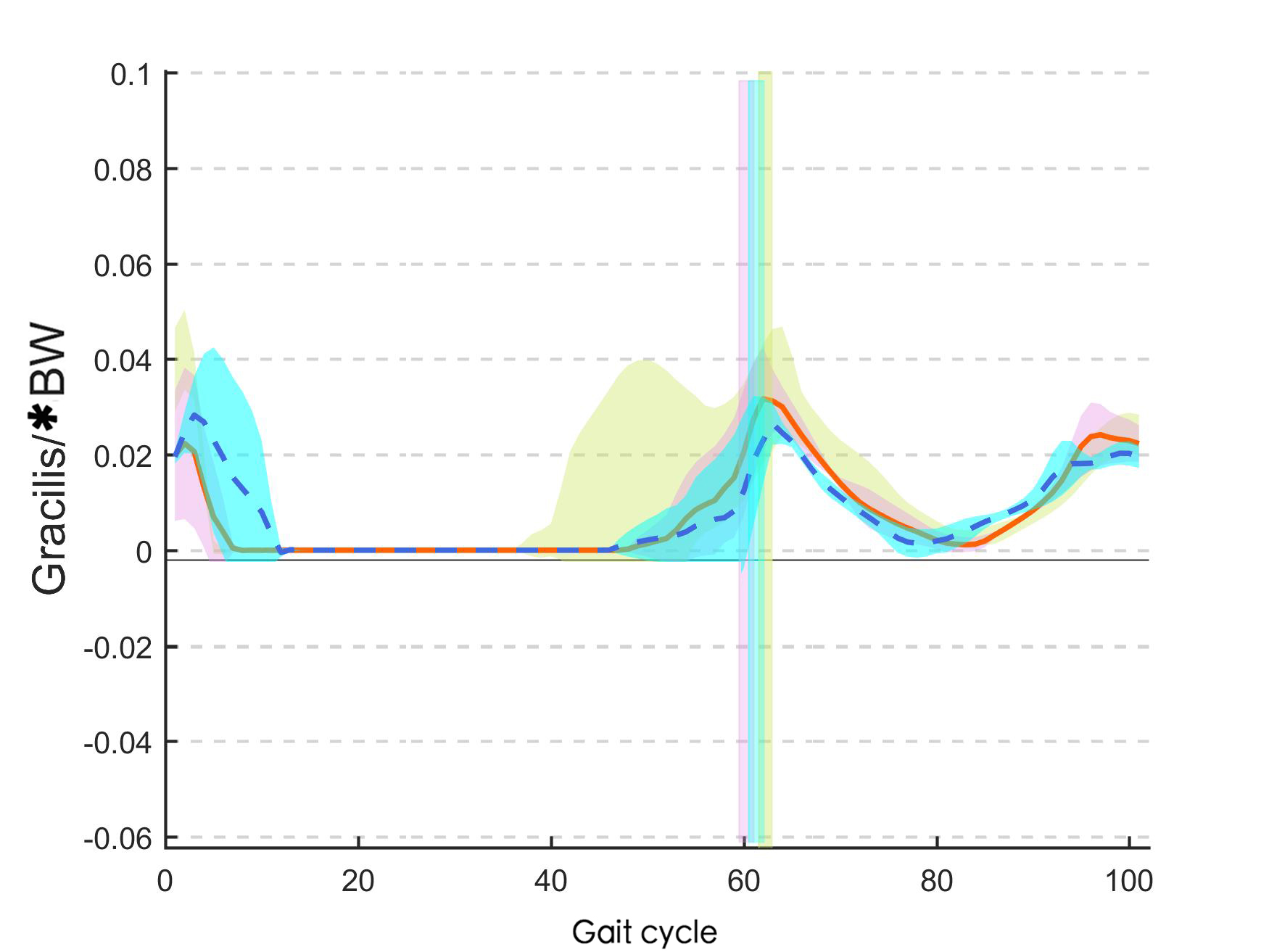

Supplement: Supplementary file 1 [file DataSheet1.ZIP › IDA RESULTS/dl57.jpg]

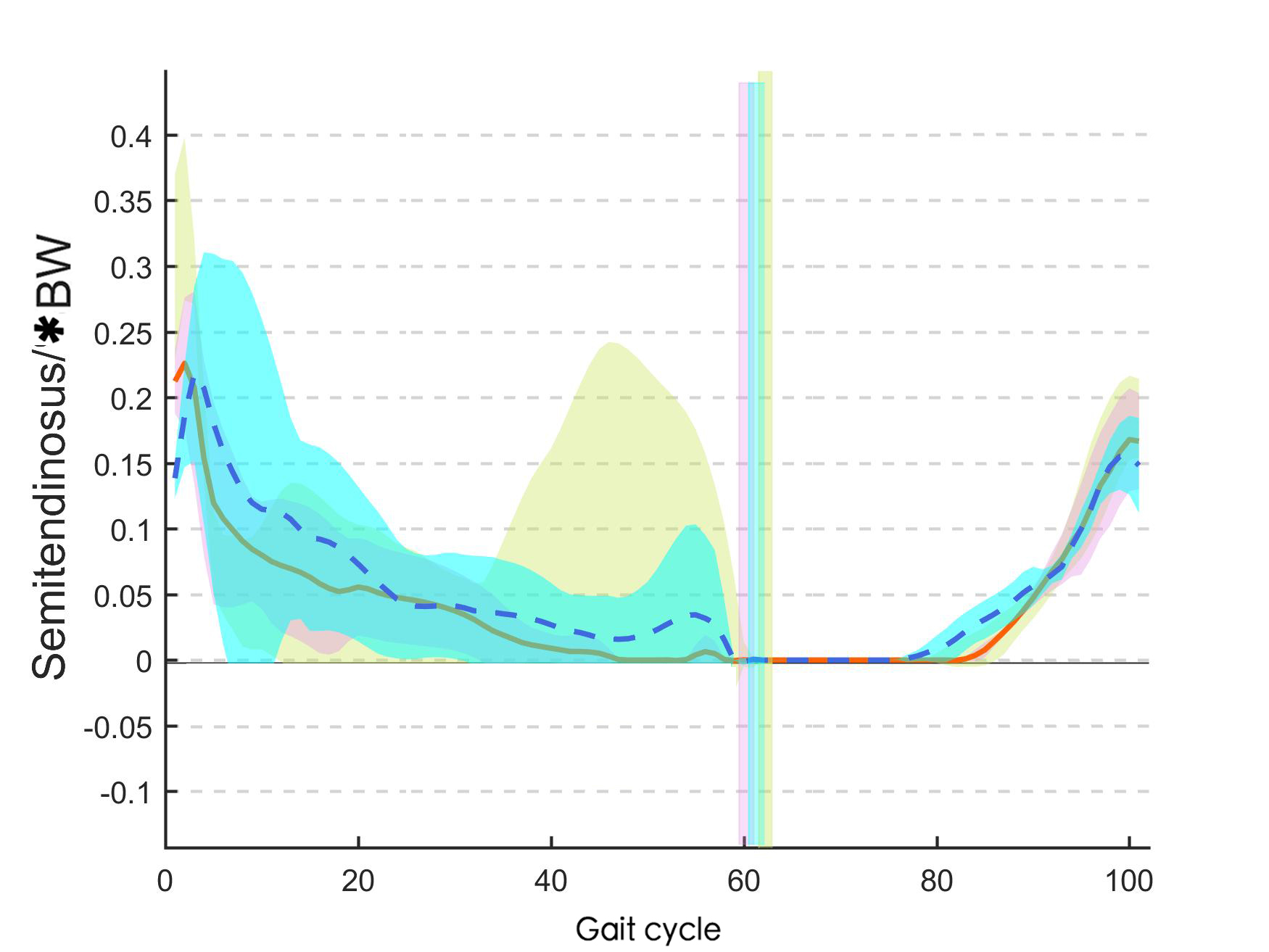

Supplement: Supplementary file 1 [file DataSheet1.ZIP › IDA RESULTS/dl58.jpg]

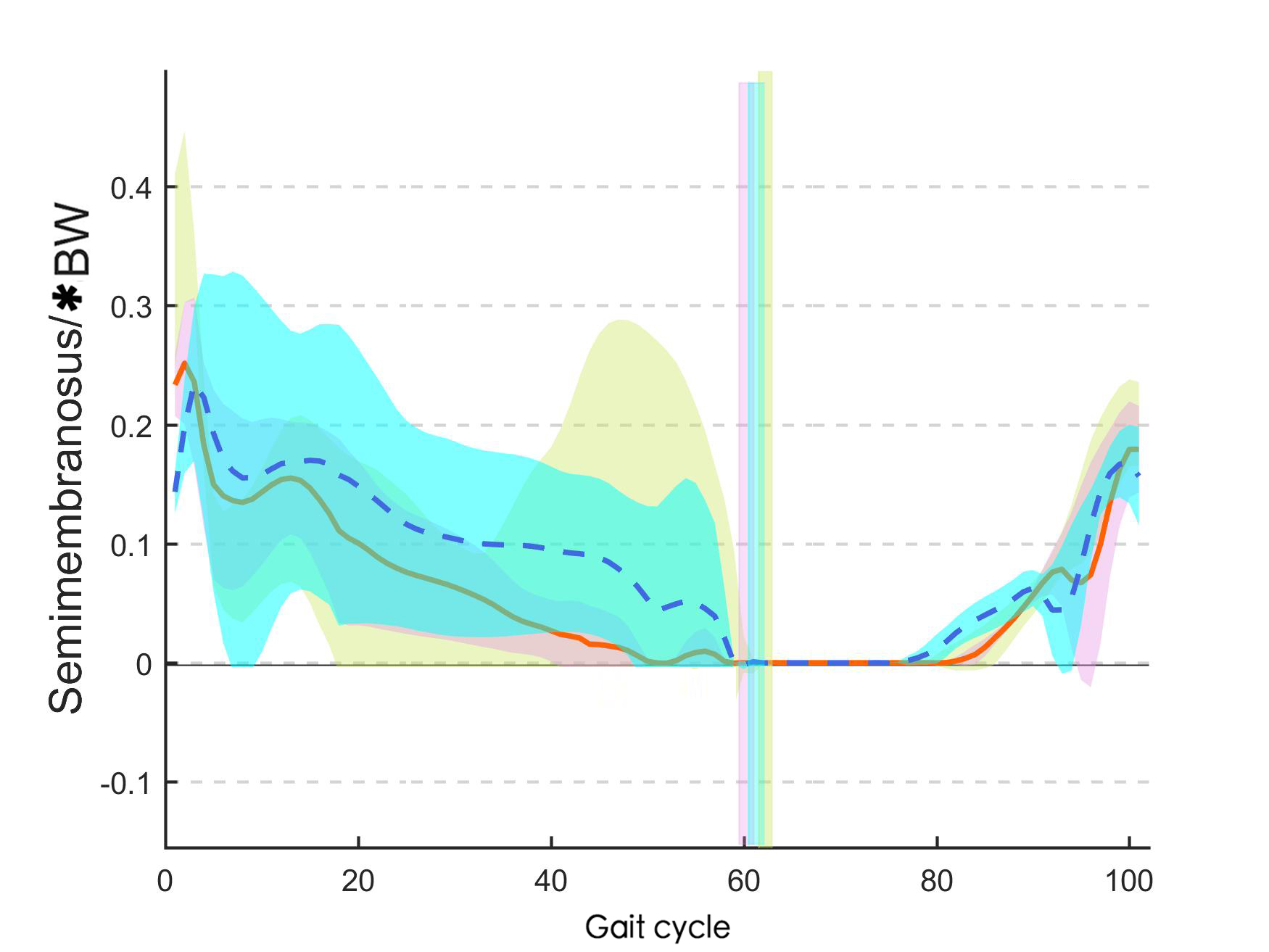

Supplement: Supplementary file 1 [file DataSheet1.ZIP › IDA RESULTS/dl59.jpg]

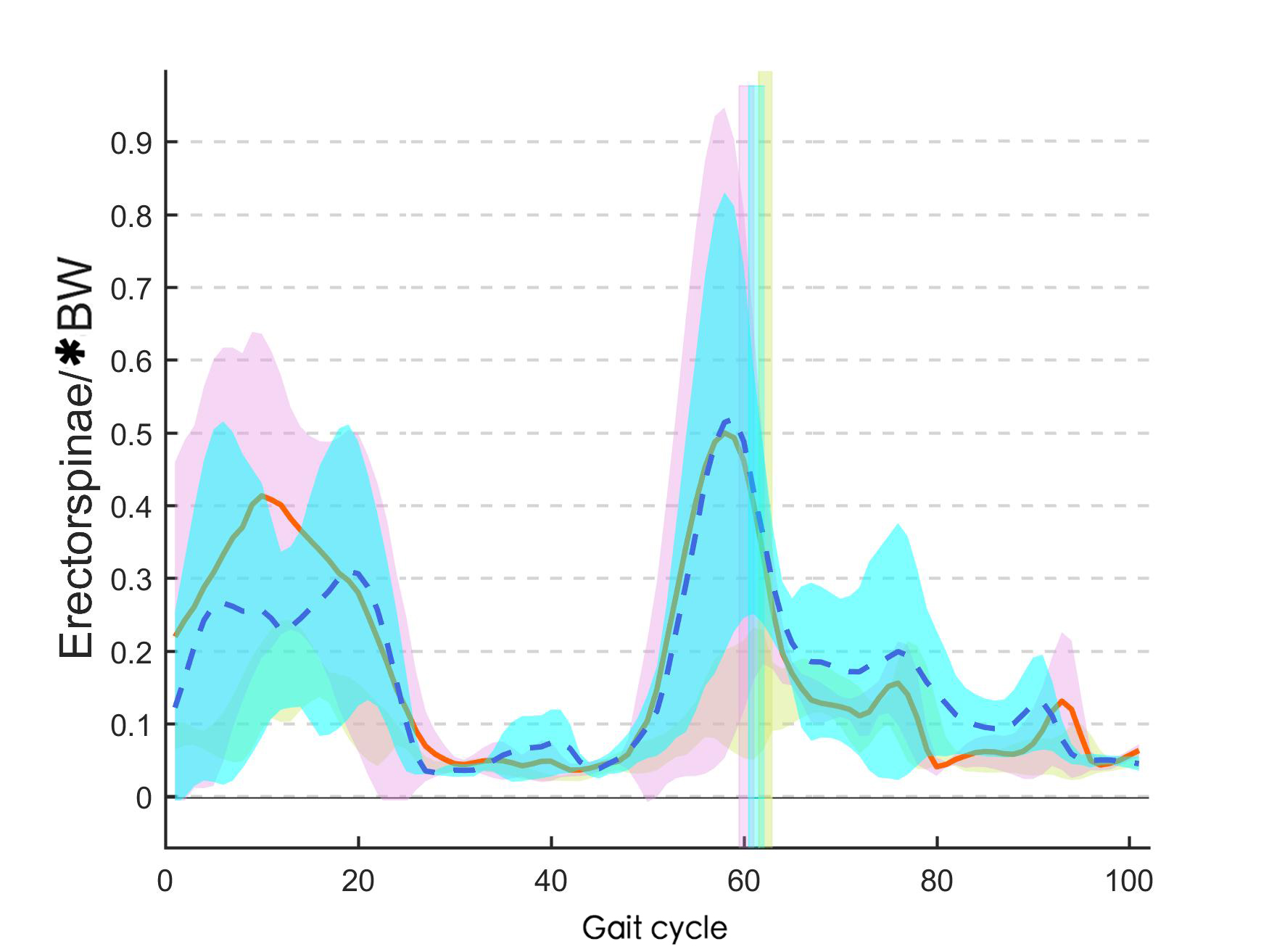

Supplement: Supplementary file 1 [file DataSheet1.ZIP › IDA RESULTS/dl60.jpg]

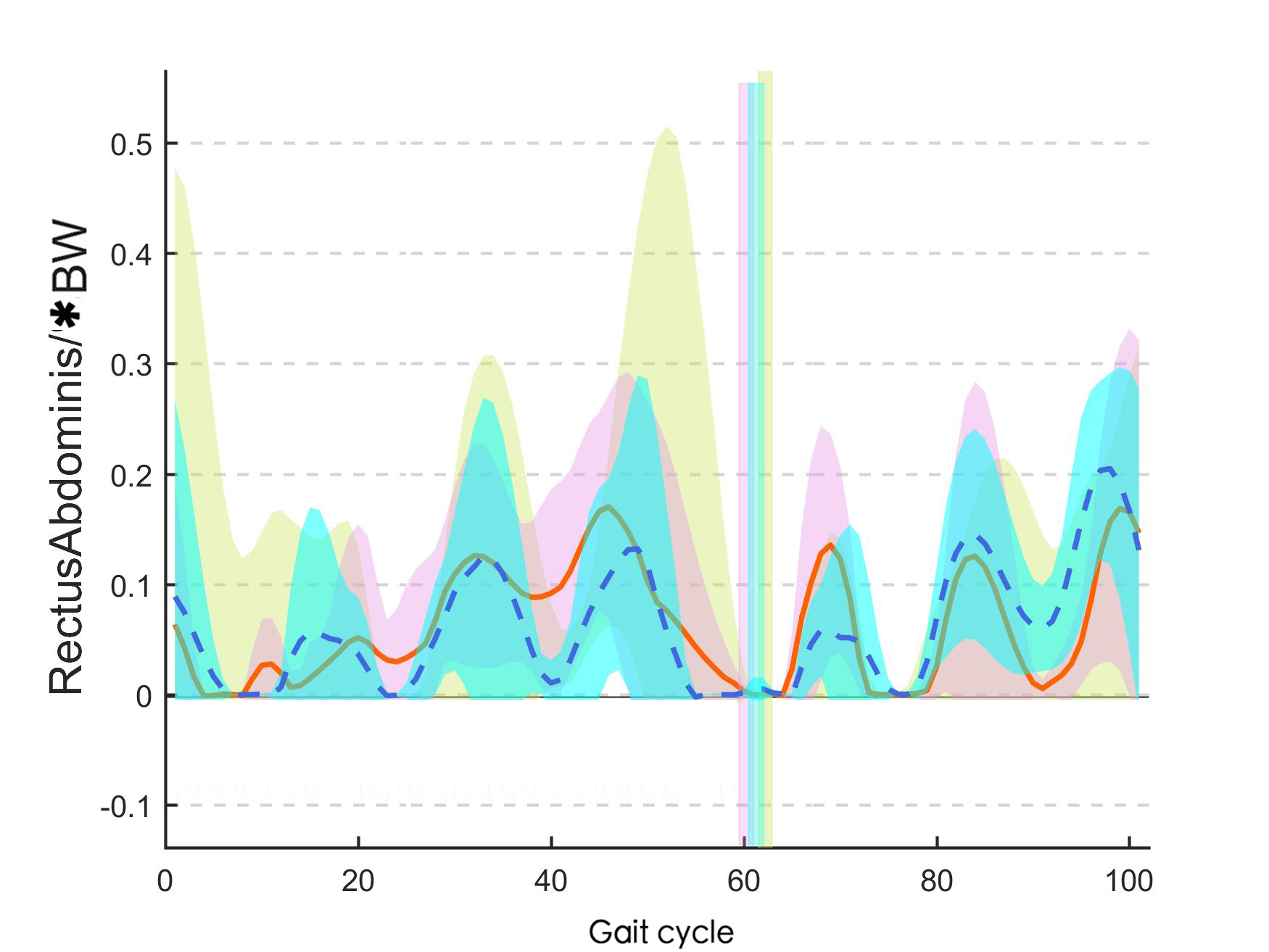

Supplement: Supplementary file 1 [file DataSheet1.ZIP › IDA RESULTS/dl61.jpg]

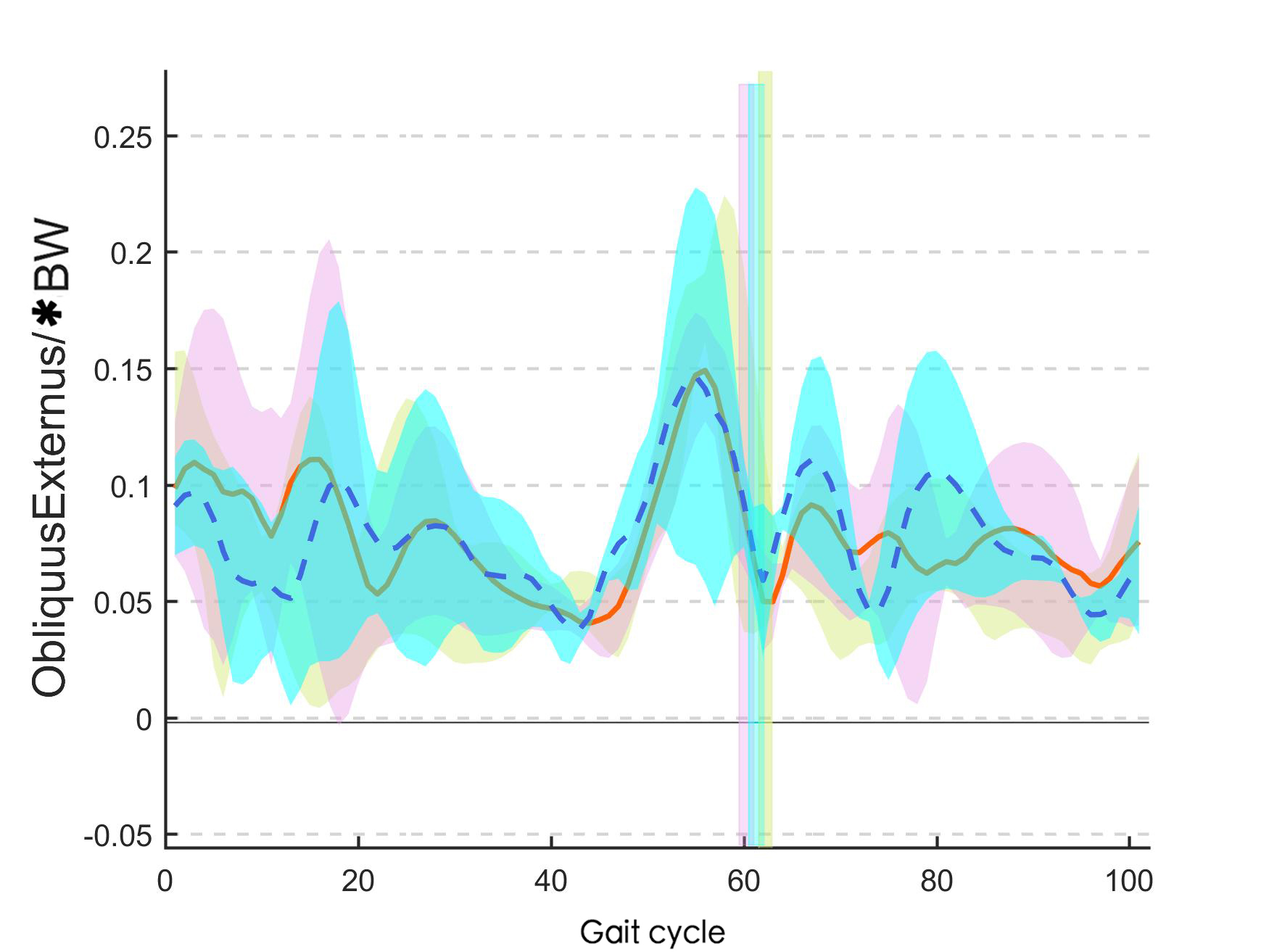

Supplement: Supplementary file 1 [file DataSheet1.ZIP › IDA RESULTS/dl62.jpg]

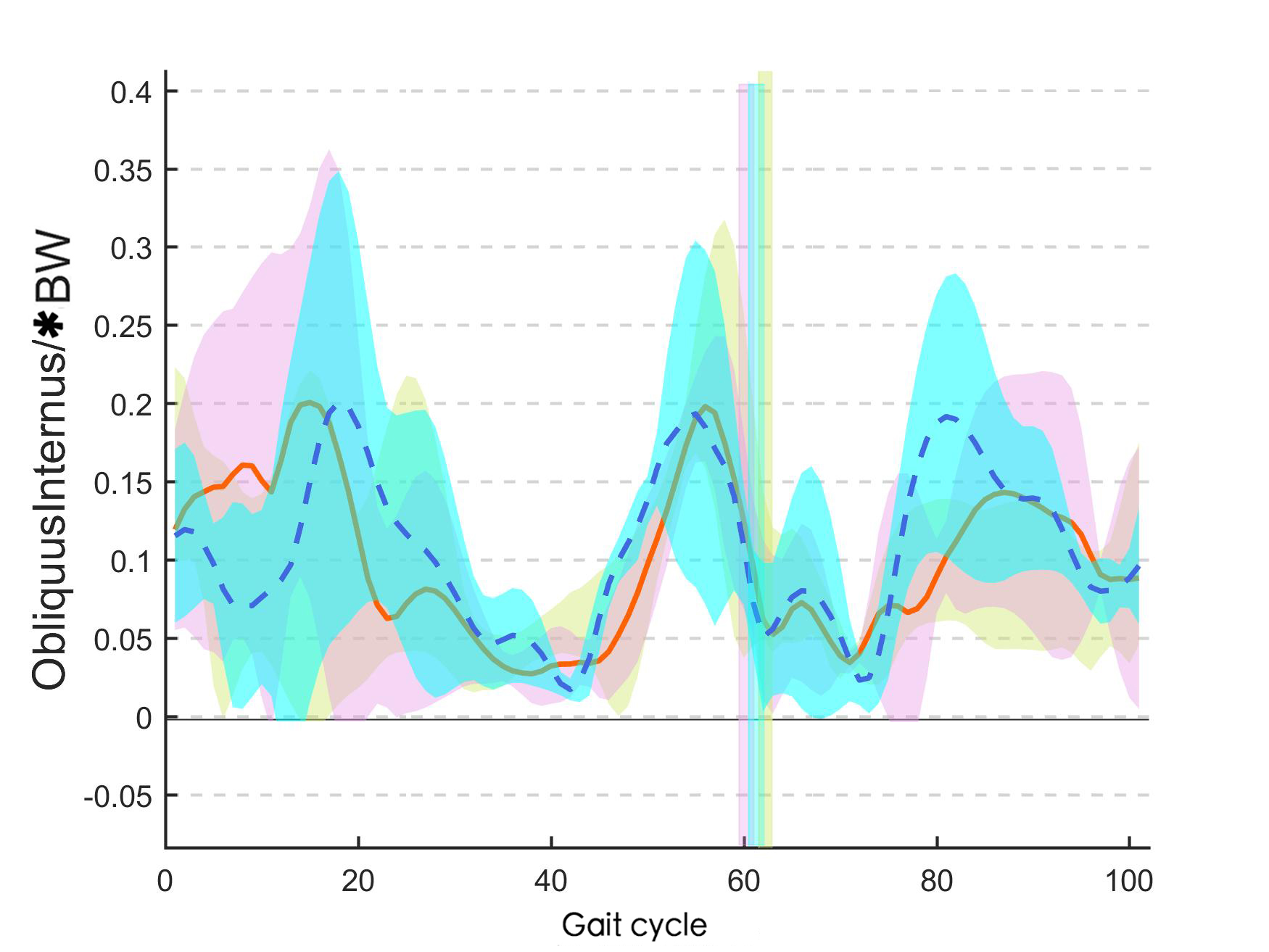

Supplement: Supplementary file 1 [file DataSheet1.ZIP › IDA RESULTS/dl63.jpg]

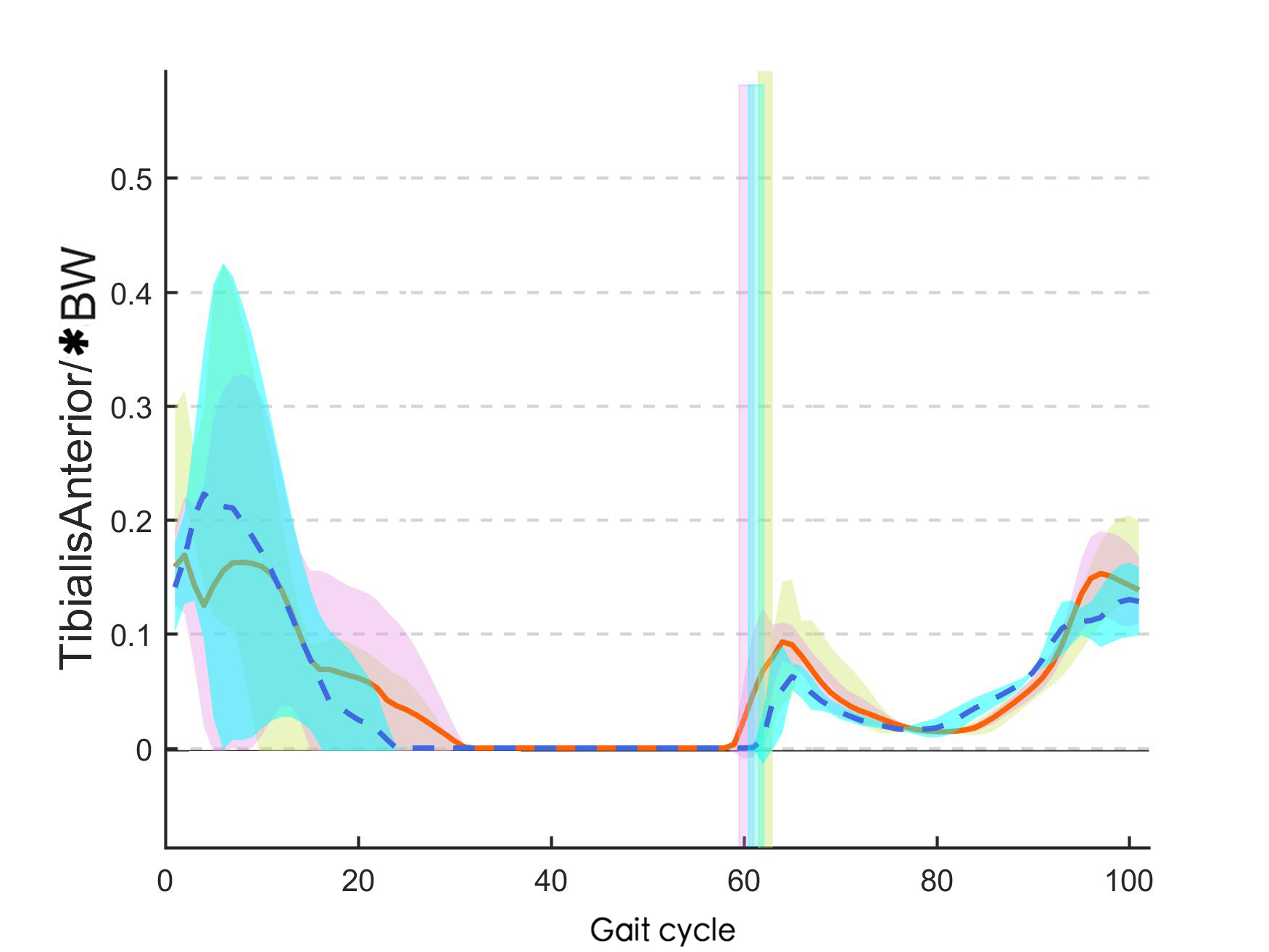

Supplement: Supplementary file 1 [file DataSheet1.ZIP › IDA RESULTS/dl64.jpg]

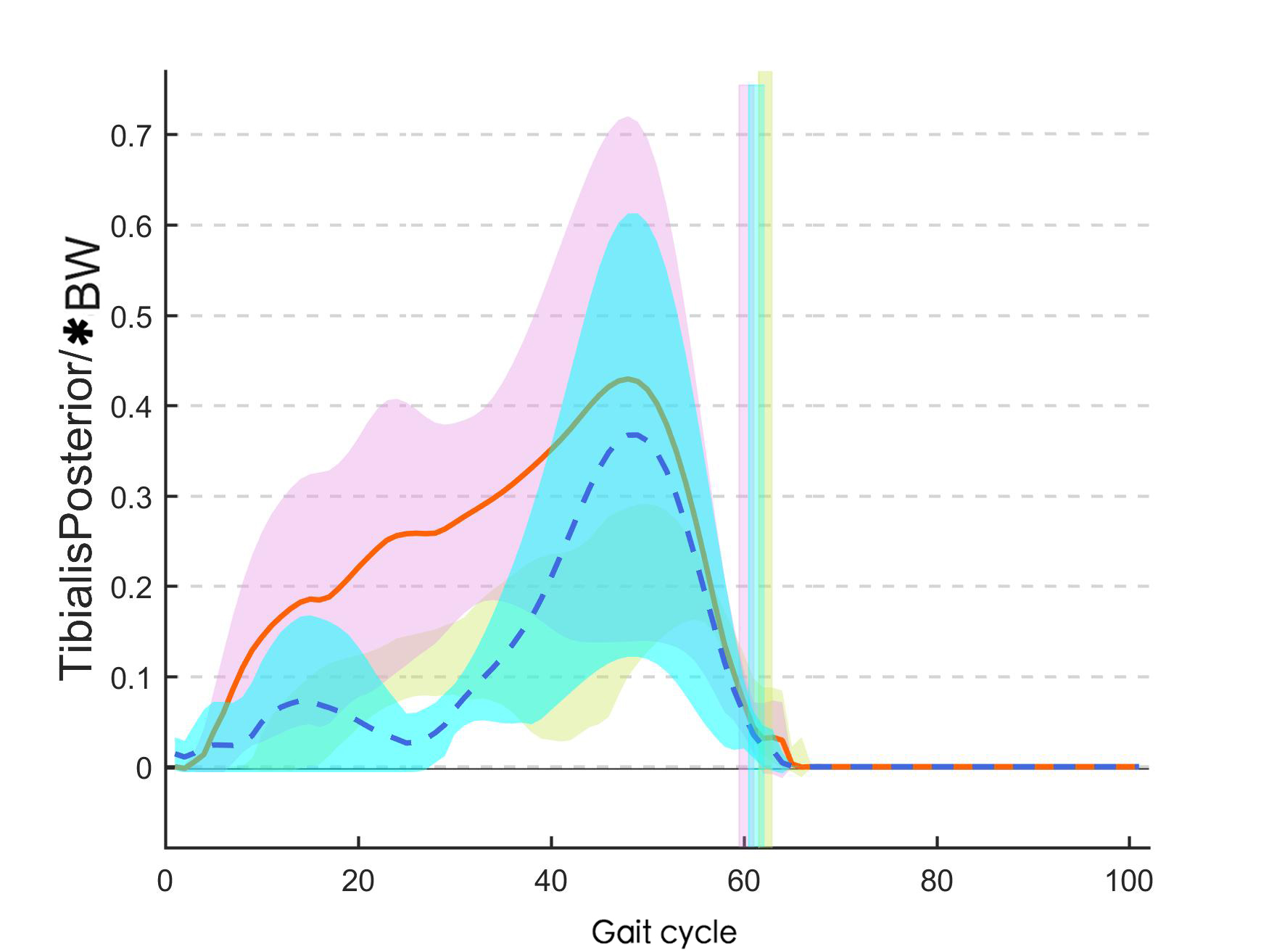

Supplement: Supplementary file 1 [file DataSheet1.ZIP › IDA RESULTS/dl65.jpg]

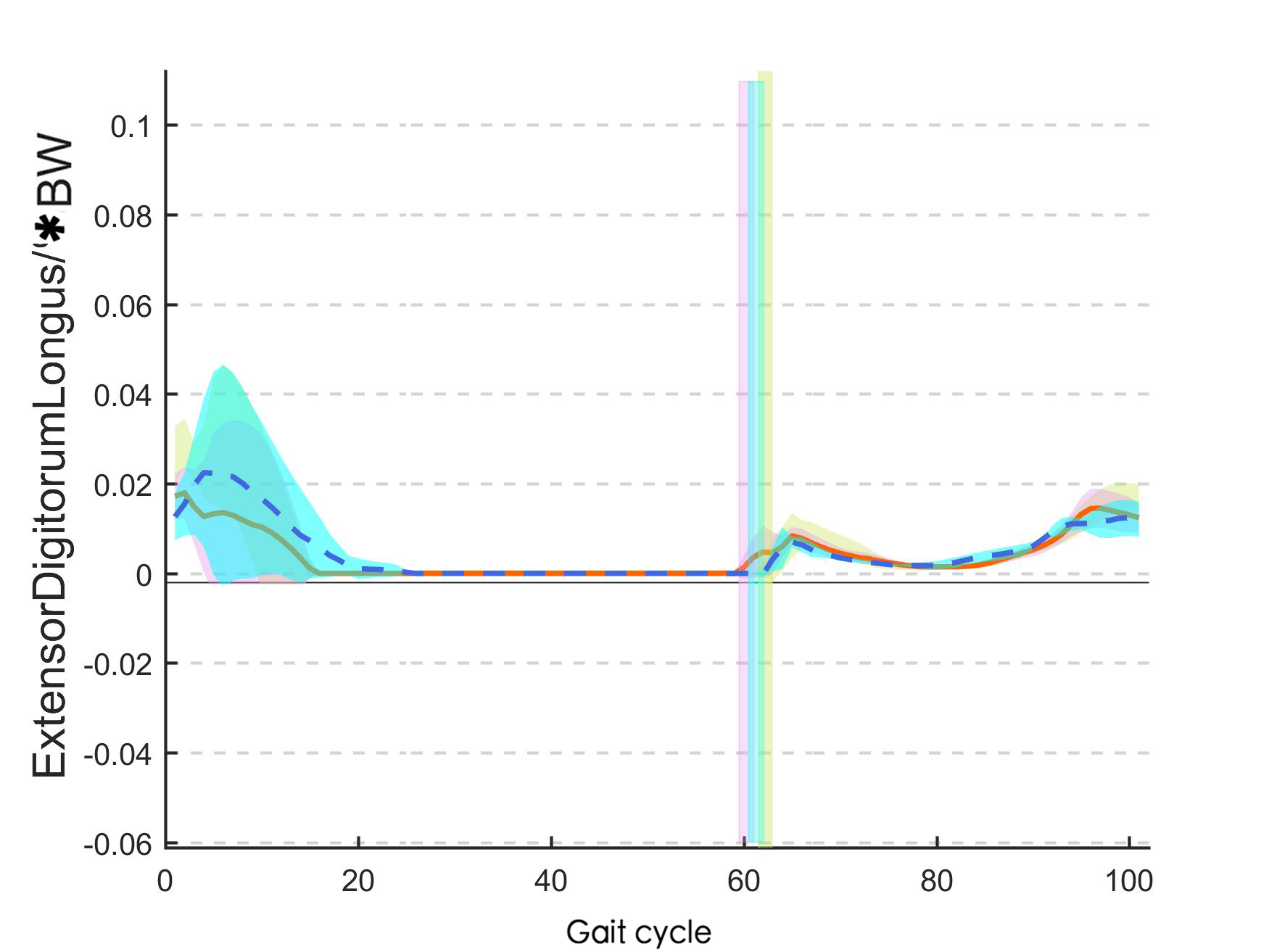

Supplement: Supplementary file 1 [file DataSheet1.ZIP › IDA RESULTS/dl66.jpg]

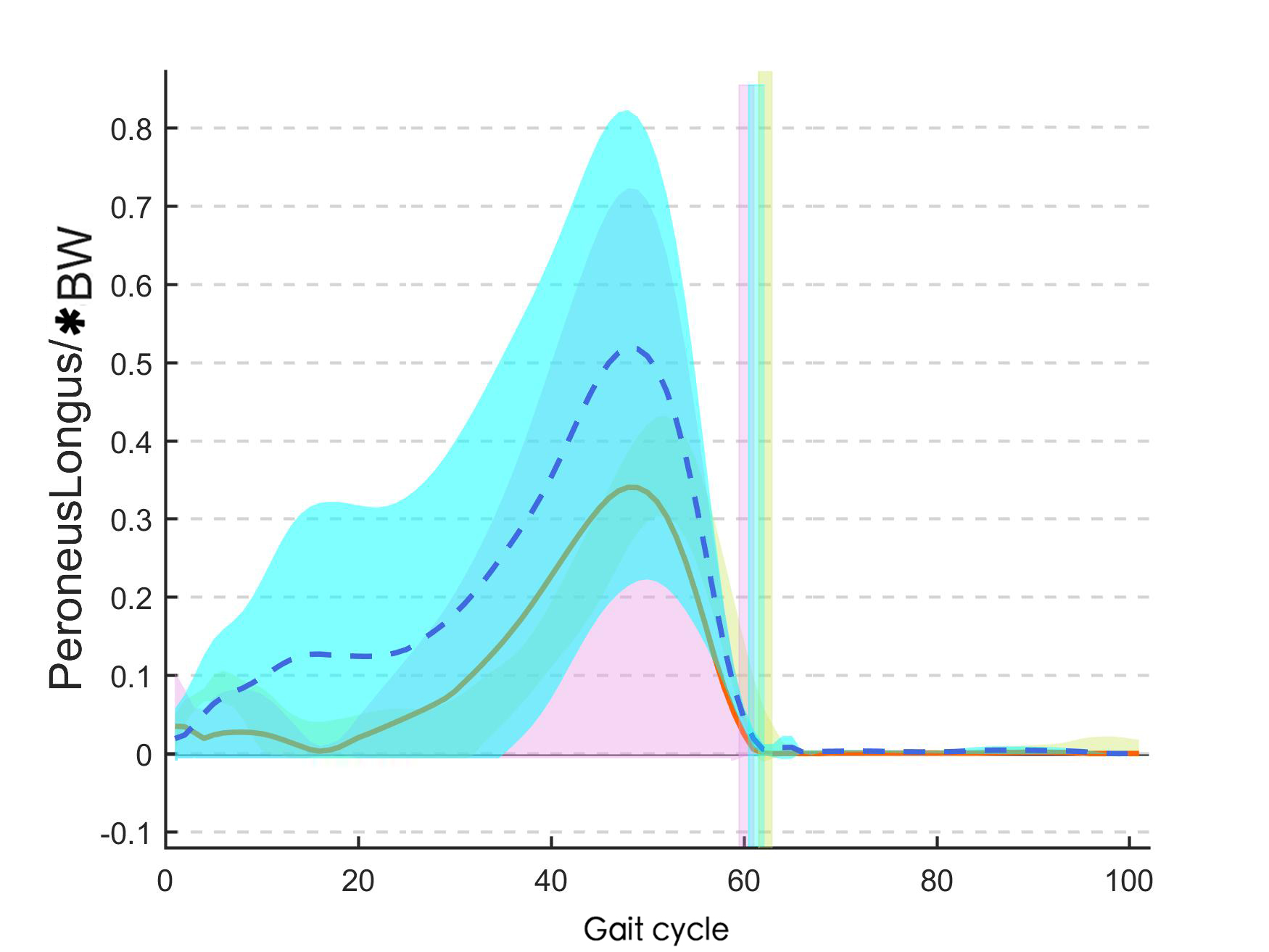

Supplement: Supplementary file 1 [file DataSheet1.ZIP › IDA RESULTS/dl67.jpg]

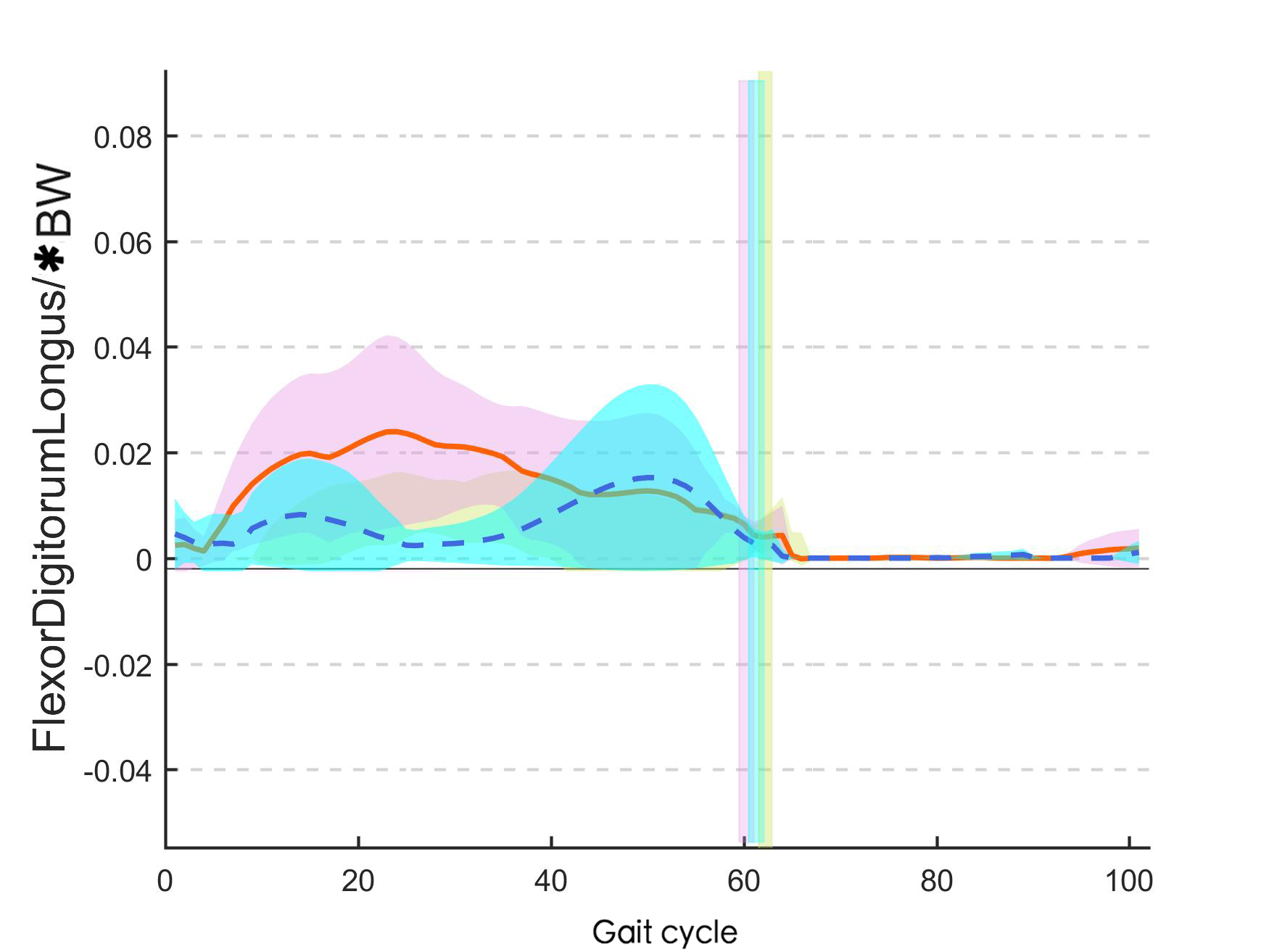

Supplement: Supplementary file 1 [file DataSheet1.ZIP › IDA RESULTS/dl68.jpg]

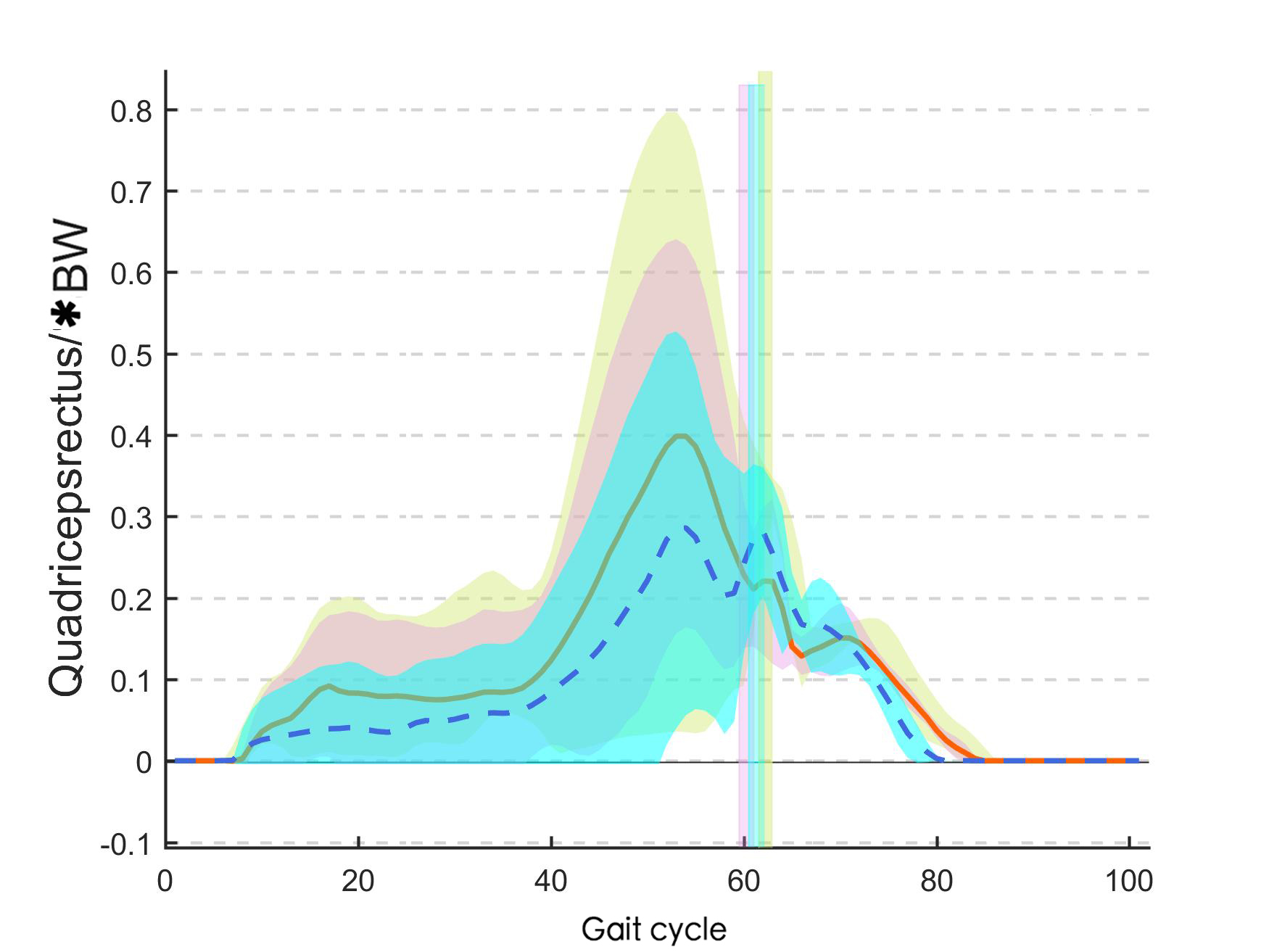

Supplement: Supplementary file 1 [file DataSheet1.ZIP › IDA RESULTS/dl69.jpg]

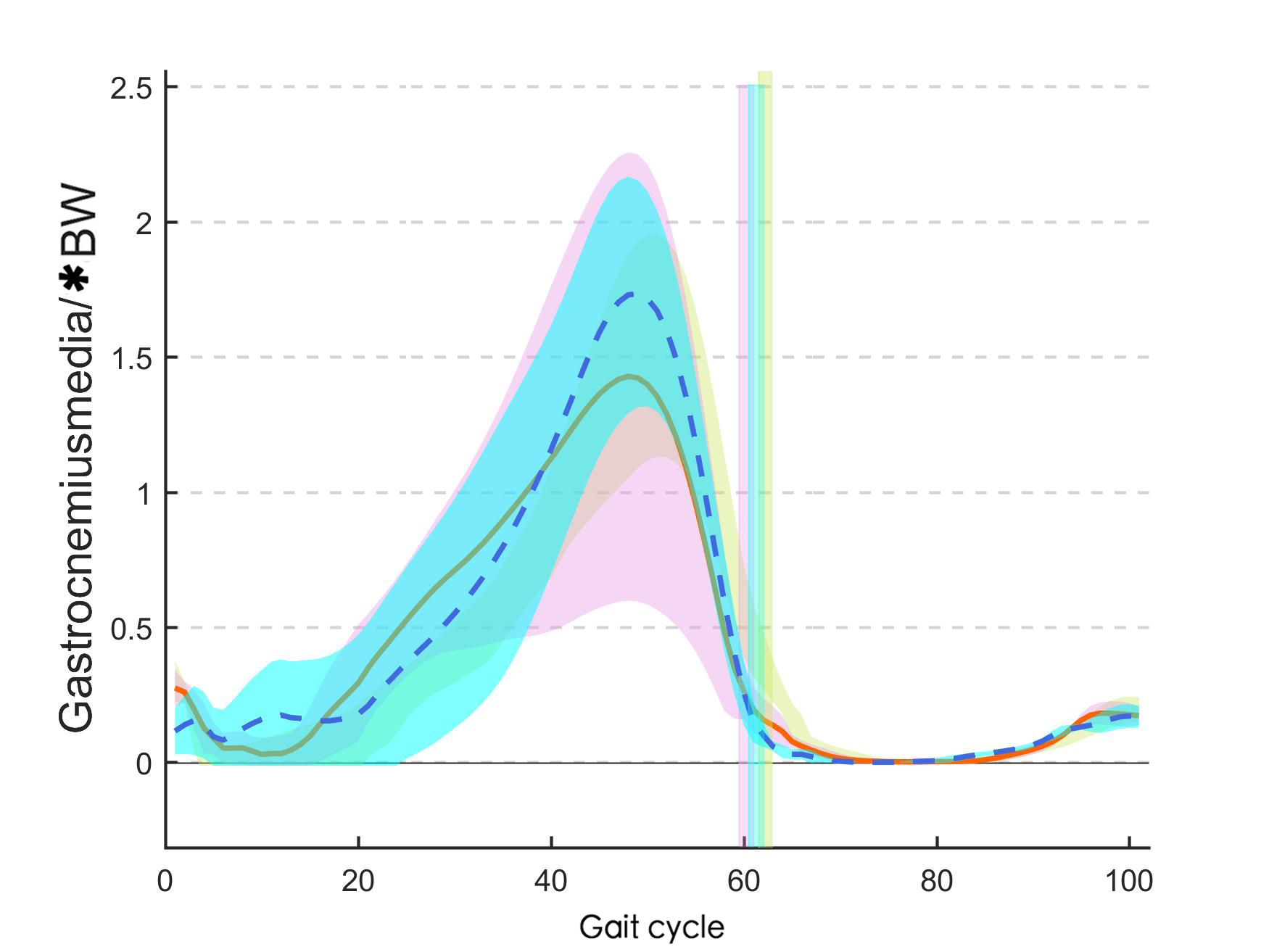

Supplement: Supplementary file 1 [file DataSheet1.ZIP › IDA RESULTS/dl70.jpg]

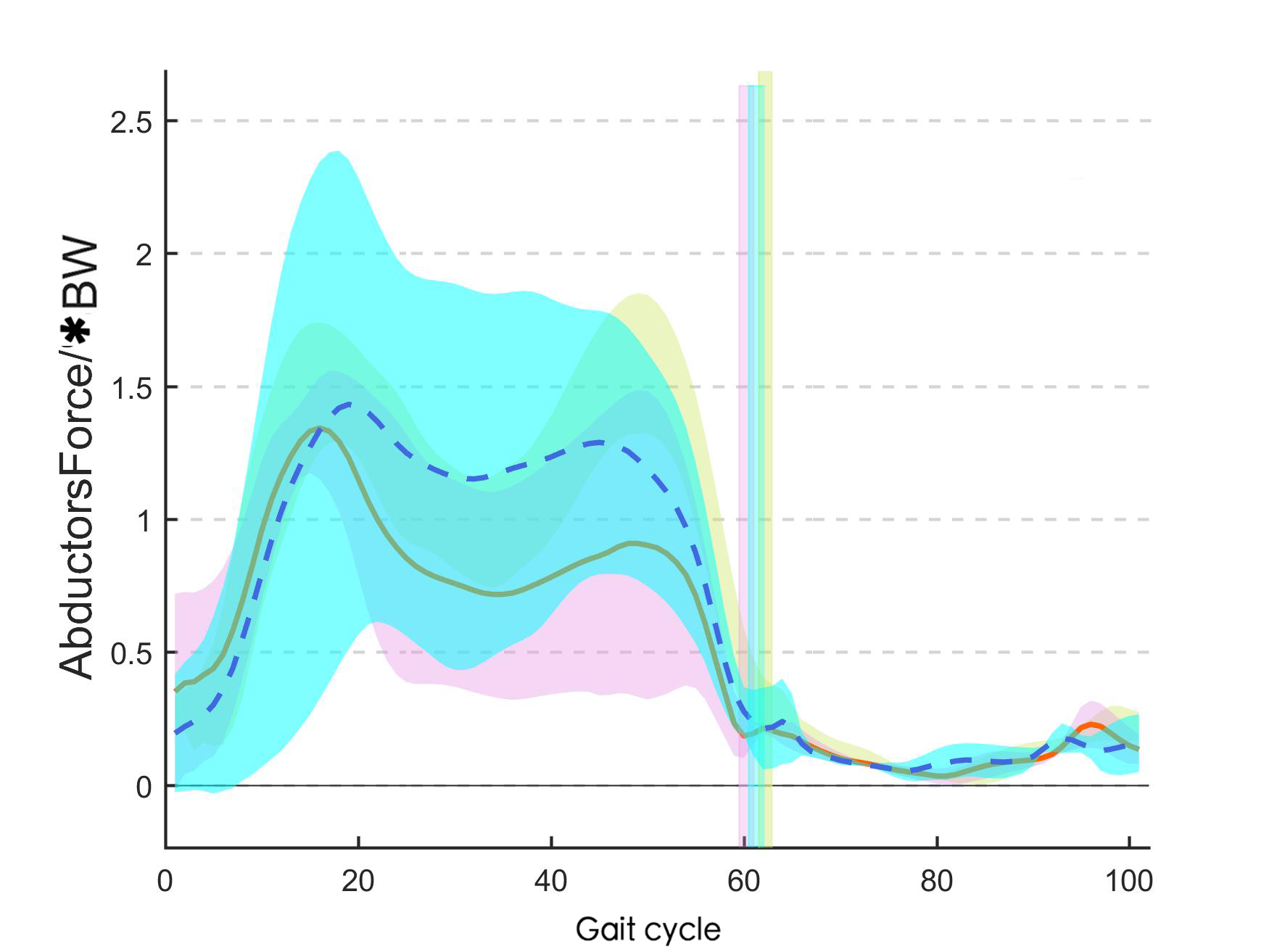

Supplement: Supplementary file 1 [file DataSheet1.ZIP › IDA RESULTS/dl71.jpg]

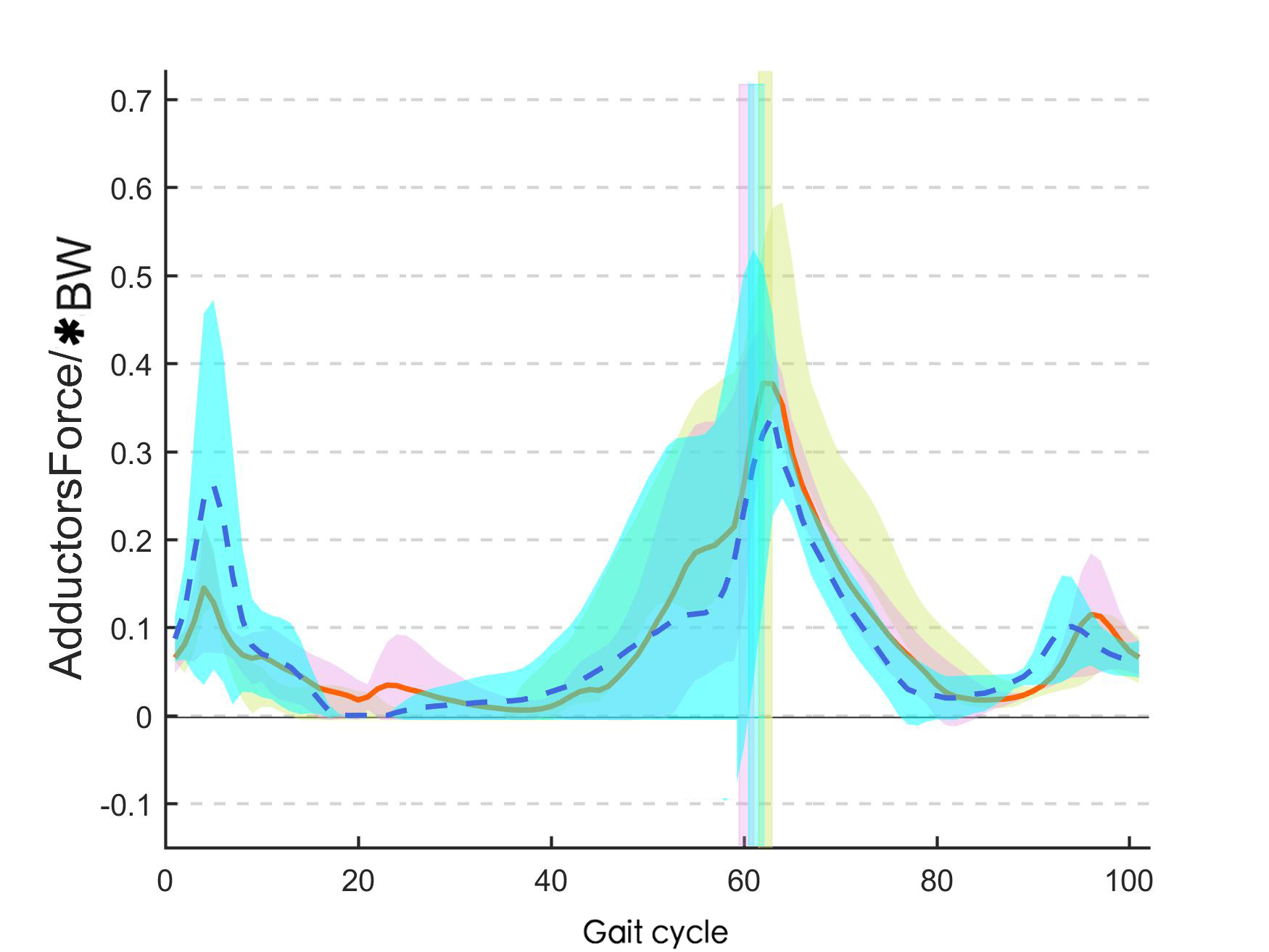

Supplement: Supplementary file 1 [file DataSheet1.ZIP › IDA RESULTS/dl72.jpg]

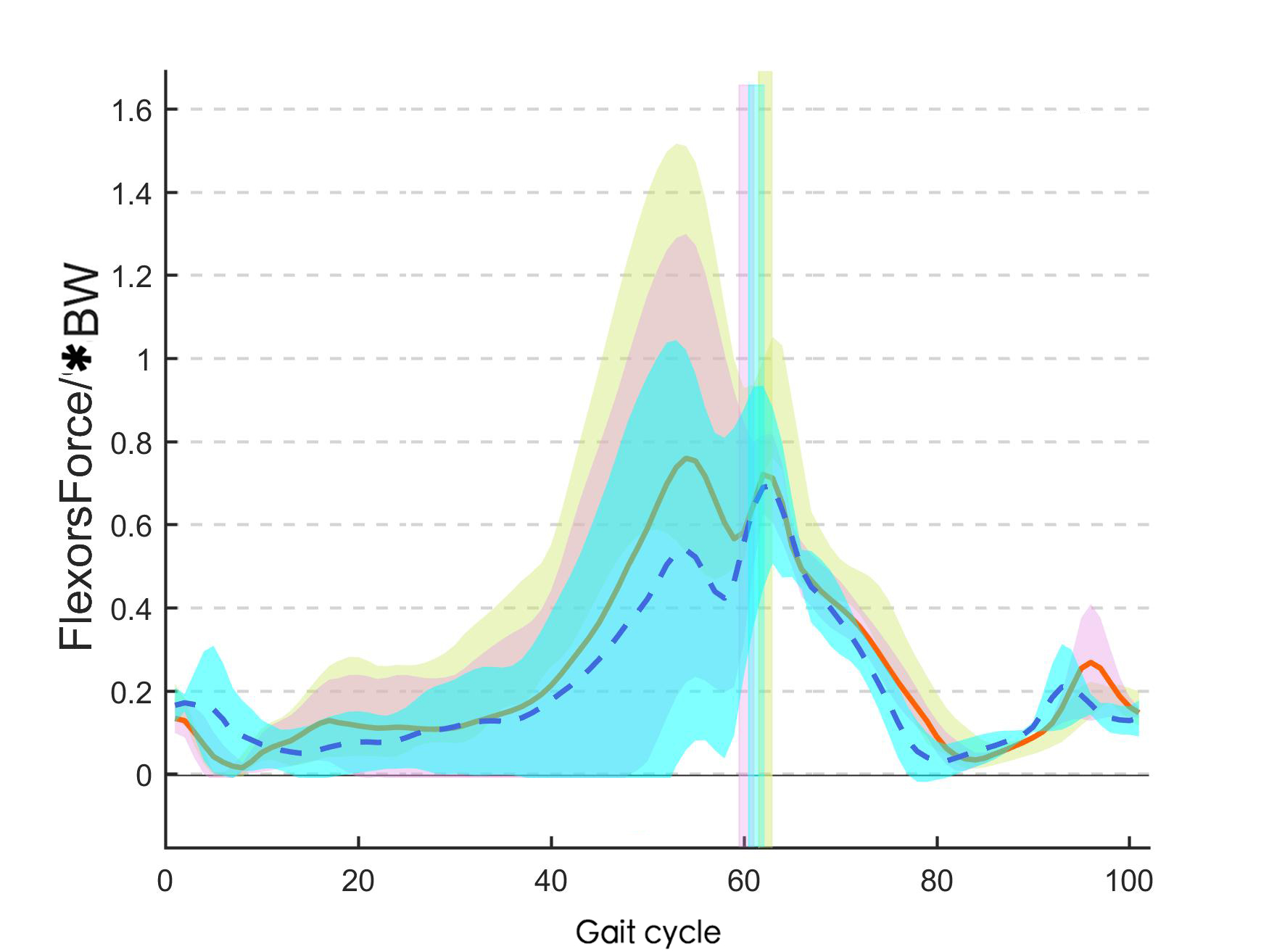

Supplement: Supplementary file 1 [file DataSheet1.ZIP › IDA RESULTS/dl73.jpg]

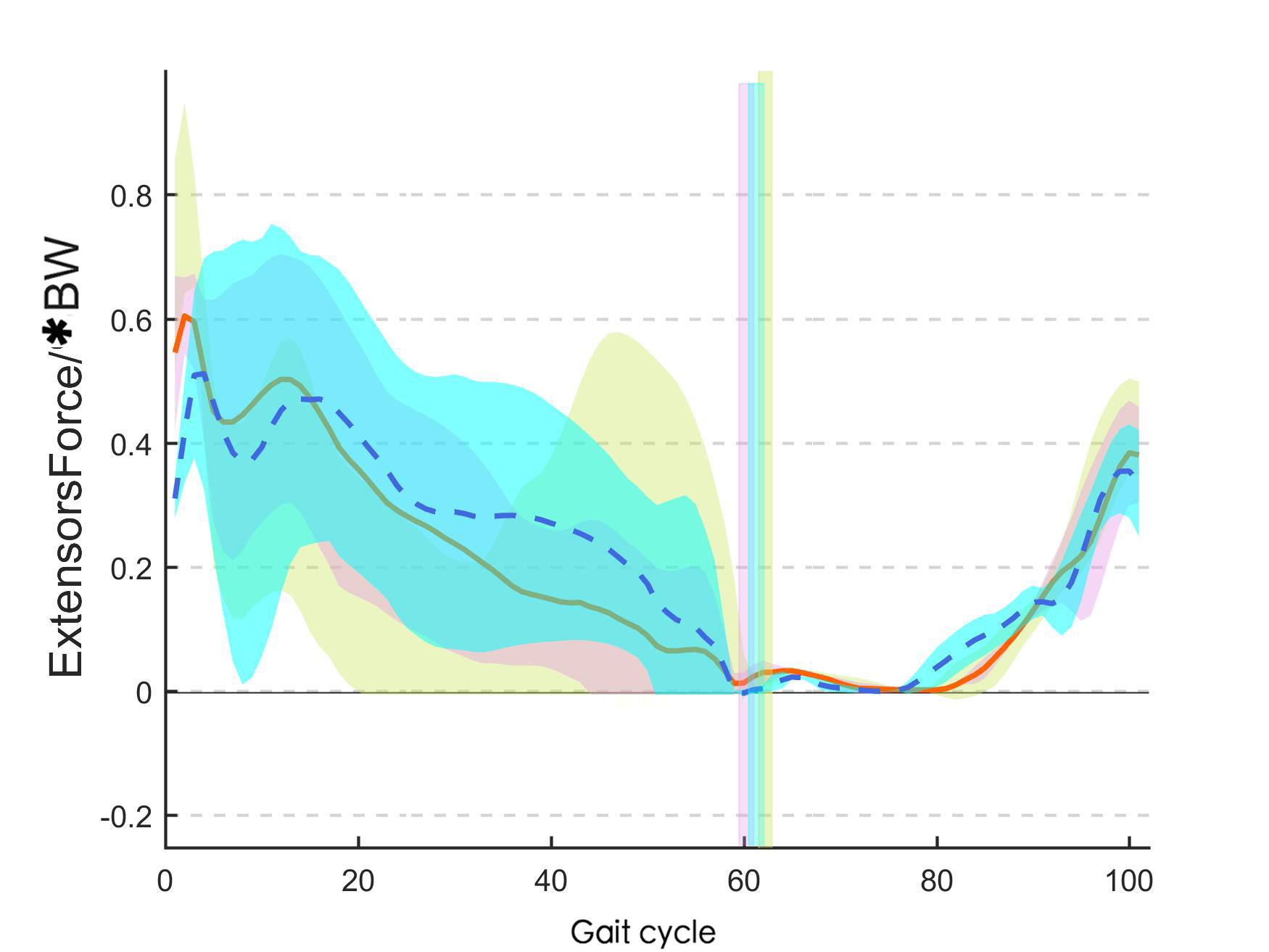

Supplement: Supplementary file 1 [file DataSheet1.ZIP › IDA RESULTS/dl74.jpg]
